# High-resolution specificity profiling and off-target prediction for site-specific DNA recombinases

**DOI:** 10.1038/s41467-019-09987-0

**Published:** 2019-04-26

**Authors:** Jeffrey L. Bessen, Lena K. Afeyan, Vlado Dančík, Luke W. Koblan, David B. Thompson, Chas Leichner, Paul A. Clemons, David R. Liu

**Affiliations:** 1 Merkin Institute of Transformative Technologies in Healthcare, Broad Institute of Harvard and MIT, Cambridge, MA 02142 USA; 2 Department of Chemistry and Chemical Biology, Harvard University, Cambridge, MA 02138 USA; 3 Howard Hughes Medical Institute, Harvard University, Cambridge, MA 02138 USA; 4 Chemical Biology and Therapeutics Science Program, Broad Institute of Harvard and MIT, Cambridge, MA 02142 USA; 5 Google Inc., Mountain View, CA 94043 USA

**Keywords:** Genetic engineering, Gene targeting, Sequencing

## Abstract

The development of site-specific recombinases (SSRs) as genome editing agents is limited by the difficulty of altering their native DNA specificities. Here we describe Rec-seq, a method for revealing the DNA specificity determinants and potential off-target substrates of SSRs in a comprehensive and unbiased manner. We applied Rec-seq to characterize the DNA specificity determinants of several natural and evolved SSRs including Cre, evolved variants of Cre, and other SSR family members. Rec-seq profiling of these enzymes and mutants thereof revealed previously uncharacterized SSR interactions, including specificity determinants not evident from SSR:DNA structures. Finally, we used Rec-seq specificity profiles to predict off-target substrates of Tre and Brec1 recombinases, including endogenous human genomic sequences, and confirmed their ability to recombine these off-target sequences in human cells. These findings establish Rec-seq as a high-resolution method for rapidly characterizing the DNA specificity of recombinases with single-nucleotide resolution, and for informing their further development.

## Abstract (web summary)

The development of site-specific recombinases as genome editing tools is limited by the difficulty of altering their DNA sequence specificity. Here the authors present Rec-seq, a method for identifying specificity determinants and off-target substrates of recombinases in an unbiased manner.

## Introduction

Site-specific recombinases (SSRs) have the potential to serve as ideal genome editing agents because they directly catalyze the cleavage, strand exchange, and rejoining of DNA fragments at defined recombination targets^1^ without relying on the endogenous repair of double-strand breaks, which can induce indels, translocations, other DNA rearrangements, or p53 activation^2–5^. The reactions catalyzed by SSRs can result in the direct replacement, insertion, or deletion of target DNA fragments with efficiencies exceeding those of homology-directed repair^1,6^. SSRs are active in a variety of cell states including non-dividing cells^1^, and many efficiently operate on mammalian genomes^7,8^. One of the most commonly used SSRs, Cre recombinase, recognizes the 34-bp *loxP* target, and is frequently used in transgenic animals for applications including conditional gene regulation^9,10^ and lineage tracing^11,12^.

Although SSRs offer many advantages, their native substrate preferences are not easily altered, even with extensive laboratory engineering or evolution^13^. Buchholz and co-workers used 126 and 145 rounds of laboratory evolution to evolve two Cre variants, Tre^14^ and Brec1 (ref. ^15^), that recombine sites differing from *loxP* at 50% and 68% of DNA base pairs, respectively. Separately, we and other researchers have begun to develop programmable recombinases by combining the capabilities of SSRs with the versatility of programmable DNA-binding proteins^16–19^. Despite continued efforts to develop SSRs, the challenges of altering their DNA specificity to manipulate arbitrary sequences of interest remains a barrier to their widespread use for genome editing.

The development of SSRs into versatile genome editing agents is limited in part by an incomplete understanding of SSR protein:DNA specificity determinants^8,13,20^. Crystal structures of tyrosine-family SSRs demonstrate that Cre and other recombinases interact with DNA through few direct protein:DNA contacts, and that shape- and charge-complementarity and water-mediated interactions contribute to SSR specificity^8,21^. Further, static co-crystal structures do not comprehensively identify key interactions between SSR residues and substrate nucleotides. For example, replacement of Glu262 increases Cre’s tolerance for mismatches in regions of *loxP* with no direct protein:DNA contacts^22^. These and other observations establish that the relationship between SSR residues and DNA specificity is not straightforward; some residues impact specificity more than others, and some contribute to specificity at distant DNA positions.

Efforts to develop programmable recombinases from existing SSRs would greatly benefit from an enhanced understanding of their DNA specificity. Motivated by this need, we sought to develop a method to rapidly map the determinants of SSR specificity. Such a method could also be used to predict cellular off-target activity of SSRs, an important consideration when evaluating SSRs as potential tools or therapeutics. Here we describe Rec-seq, a method for profiling the DNA specificity of SSRs in a rapid and unbiased manner using in vitro selection and high-throughput DNA sequencing (HTS). We applied Rec-seq to characterize wild-type Cre and Cre mutants, resulting in the identification of novel DNA specificity determinants, including long-range interactions not evident from structural studies. We profiled the laboratory-evolved Cre variants Tre and Brec1, as well as three orthogonal SSRs, including the integrase Bxb1. The application of Rec-seq to Tre and Brec1 recombinases resulted in specificity profiles that accurately predicted activity at off-target sites, including pseudosites within the human genome. Our findings suggest that Rec-seq can inform the application of SSRs as well as their further development.

## Results

### An in vitro selection for recombinase substrates

**Fig. 1:**
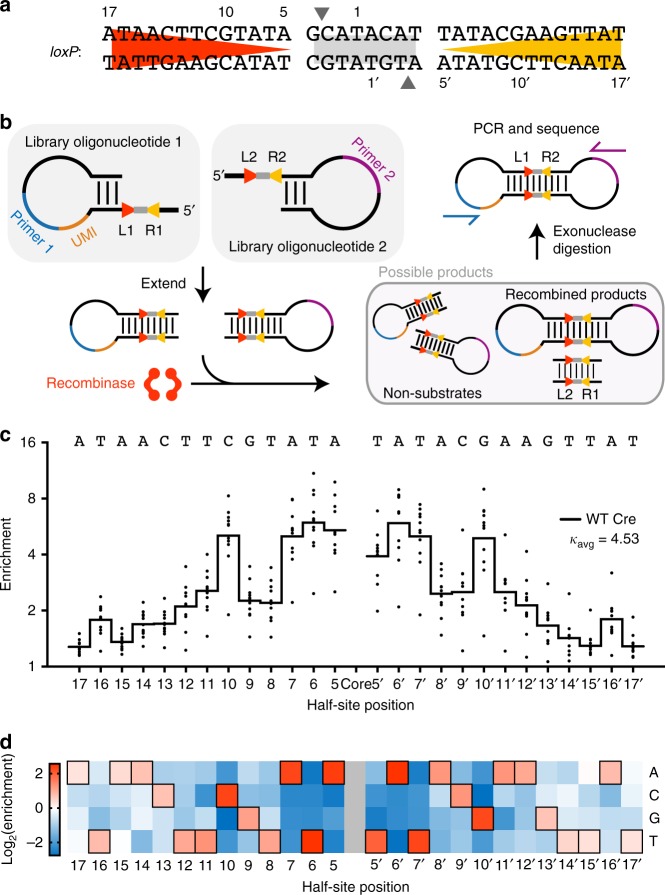
Recombinase specificity profiling of wild-type Cre. **a** The cognate DNA substrate of Cre, *loxP*. DNA backbone cleavage occurs at the indicated phosphodiester bond (gray arrows). **b** Overview of Rec-seq. DNA hairpin oligonucleotides containing partially randomized *loxP* sites and a unique molecular identifier (UMI) are subjected to intramolecular primer extension, exposed to recombinase, and digested with exonucleases to destroy non-recombined DNA. **c** The specificity profile for Cre shows its relative preference for the canonical base at each position in the *loxP* site. The quality score *κ*_avg_ represents the number of unique recombination events captured by Rec-Seq across each experimental replicate, with a value over 1.5 considered a well-powered experiment. Values represent the geometric mean of *n* = 11 independent replicates (dots) conducted at 37 °C for 30 min with a 1:3 ratio of total moles of recombinase:total moles of DNA molecules. **d** Heat map of Rec-seq enrichment values for wild-type Cre showing the log_2_ of the enrichment value for each nucleotide at each position in *loxP* relative to the canonical base (black outline). Source data are provided as a Source Data file

We sought to develop a system for profiling recombinase specificity through identification of bona fide recombinase substrates from a vast in vitro library of possible targets. To do so, we designed substrate oligonucleotides such that recombination yields a degradation-resistant DNA product, permitting the selective digestion of non-substrates. We chose Cre as a model recombinase for developing Rec-seq because Cre has been structurally characterized^8^, the effects of some Cre mutations on DNA specificity are known^21–28^, and researchers have generated Cre variants with altered specificity^13^. Cre’s substrate *loxP* consists of two 13-bp half-sites that together form inverted repeats, flanking an asymmetric 8-bp core region where strand exchange occurs (Fig. 1a).

To prepare in vitro substrate libraries, we extended synthetic DNA containing self-priming 5′ overhangs and a partially randomized *loxP* site (Fig. 1b). The hairpin serves to prime extension across the randomized region of *loxP*, replicating the library member and yielding a double-stranded DNA substrate required by SSRs. We generated two related substrates: left-hairpin substrates (containing left and right half-sites L1 and R1) and right-hairpin substrates (containing half-sites L2 and R2; Fig. 1b). When Cre protein is exposed to one left-hairpin and one right-hairpin oligonucleotide, successful recombination generates a DNA product with hairpins on both sides. Exonuclease treatment destroys non-recombined library members, and the exonuclease-resistant double-hairpin recombination products are amplified by PCR. HTS of libraries (at a typical depth of 10^5^–10^6^ reads per experiment) enables quantitation of the frequency of each base at each half-site position before and after selection. Enrichment scores are then determined for each target position (Supplementary Note 1), such that higher enrichment scores reflect a stronger preference for a particular base at that half-site position.

In designing the Rec-seq library we considered the optimal degree of *loxP* randomization and the ideal placement of these randomized positions within the Rec-seq oligonucleotides. Since Cre is thought to be highly specific for *loxP*, we hypothesized that a modest number of mutations per half-site would support recombination while allowing the interrogation of many substrate combinations. Randomized positions in *loxP* were varied during DNA synthesis to contain 79% wild-type base and 21% of an equimolar mixture of all three other bases, yielding a library in which each variable half-site contained 2.7 mutations on average. We routinely generated libraries exceeding 10^11^ sequences, sufficient to cover all possible half-sites with up to seven substitutions from the *loxP* sequence. We found no significant differences of enrichment values when performing Rec-seq experiments with a more highly mutagenized *loxP* library (Supplementary Fig. 1). Additionally, the core sequence of *loxP* was held constant because the core regions of two recombining *loxP* substrates must be complementary^29^. Most Cre:*loxP* interactions are thought to involve the half-sites^8,22^, and we observed minimal preference among the core nucleotides in experiments in which the half-sites were held constant and the core was mutagenized (Supplementary Fig. 2). Finally, Rec-seq only captures mutations present in L1 and R2, because the product of recombination containing R1 and L2 is degraded (Fig. 1b). In order to isolate interactions between Cre and a single *loxP* half-site, only L1 or R2 was randomized while R1 and L2 were fixed as the wild-type *loxP* sequence. Enrichment profiles for a full *loxP* target were generated by collecting the enrichment factors from L1 and R2 half-sites.

Next we optimized and validated Rec-seq experimental conditions using wild-type Cre. The Cre specificity profile did not substantially change upon incubation times longer than 30 min (Supplementary Fig. 3a). A protein:DNA ratio of 1:3 was previously shown to be optimal for recombination^30^, and we found that protein:DNA ratios higher than ~1:1 eroded apparent specificity, consistent with excess enzyme enabling the recombination of even non-preferred substrates (Supplementary Fig. 3b). Finally, we showed that the Rec-seq enrichment pattern of Cre protein exposed to *loxP* substrate was not dependent on the source of Cre protein (Supplementary Fig. 4).

Before analyzing the resulting enrichment profile, we calculated a quality score for each experiment. Poorly active recombinases or very short exposure to enzyme could result in levels of bona fide substrates surviving selection that do not greatly exceed background levels of undigested library material (Supplementary Fig. 5a). To identify such instances of poor signal:background ratios, we calculated a quality score, *κ*, for each experiment. Background amplification for each experiment was measured using quantitative PCR to confirm that SSR-treated samples contained more DNA after selection than a control sample lacking recombinase. To distinguish low activity from poor specificity, we included a unique molecular identifier (UMI) barcode on the left-hairpin library member (Fig. 1a). The *κ* value for each experiment was determined by plotting the percent abundance of each DNA sequence variant in the post-recombination library versus the number of UMIs for each sequence variant, with *κ* being the slope of the best-fit line, divided by 10^4^ for ease of comparison (Supplementary Fig. 5b). The average *κ* value among experimental replicates for a given SSR, *κ*_avg_, reflects whether its Rec-seq enrichment values are derived from a large number of independent recombination events (a larger *κ*_avg_ value) or may be subject to undersampling due to low activity (a smaller *κ*_avg_ value; Supplementary Table 1). By comparing Rec-seq outcomes between experimental replicates, we considered experiments to be well-powered if *κ*_avg_ values exceeded 1.5, modestly influenced by background signal for *κ*_avg_ values between 1.5 and 0.5, and heavily influenced by background signal for *κ*_avg_ values below 0.5 (Supplementary Fig. 5c).

Analysis of the Rec-seq enrichment profile for Cre indicated a preference for the canonical base at every half-site position (Fig. 1c), a surprising finding given the limited direct protein:DNA contacts between Cre and several regions of *loxP*^8^. On average, 22% of post-selection sequences were identical to *loxP*, compared to 6.4% *loxP* abundance pre-selection. Rec-seq revealed the sequence preference of Cre to be asymmetric, as is evident when the left and right half-site enrichment profiles are superimposed (Supplementary Fig. 6a). To ensure that an asymmetric sequence preference is a property of the enzyme and not due to the different DNA sequences flanking the library oligonucleotides (Fig. 1b), we performed Rec-seq using a substrate library identical to the original except that the non-palindromic *loxP* core was replaced with its reverse complement (Supplementary Fig. 6b). The Rec-seq enrichment profile of this “inverted core” *loxP* library mirrored, rather than duplicated, the profile on the original substrate library (Supplementary Fig. 6b), indicating that the oligonucleotide sequence context was not responsible for the asymmetry of the Cre specificity profile. These findings establish the utility of Rec-seq for illuminating DNA-recognition properties of Cre that are difficult or impossible to infer solely by structural characterization.

Rec-seq also confirmed previous findings^8^ that Cre has a pronounced preference in two regions of *loxP*: half-site positions 5–7 and 10. We observed 5.0-fold enrichment of the canonical base at position 10, consistent with reports that Arg259 participates in hydrogen bonding with the C•G base pair at position 10 (refs. ^23,31^) (Fig. 1c). Rec-seq also identified a 3.9- to 5.4-fold enrichment for the canonical base pair at position 5 in each half-site, consistent with direct interactions between Gln90 and the A•T base pair^23,31^. A final notable interaction at the Cre-DNA interface is between Lys244 and the T•A base pair at positions 16–17, the only direct contact between Cre and the five most distal bases of *loxP*^31^. Indeed, among positions 13–17, Rec-seq revealed the strongest preference to be at position 16 (Fig. 1c). Together, these results validate that Rec-seq can identify DNA sequence preferences consistent with known Cre:*loxP* interactions and provide novel context to these preferences, such as the relative specificity of Cre for nucleotides in *loxP*.

### Mutational dissection of Cre:loxP specificity determinants

**Fig. 2:**
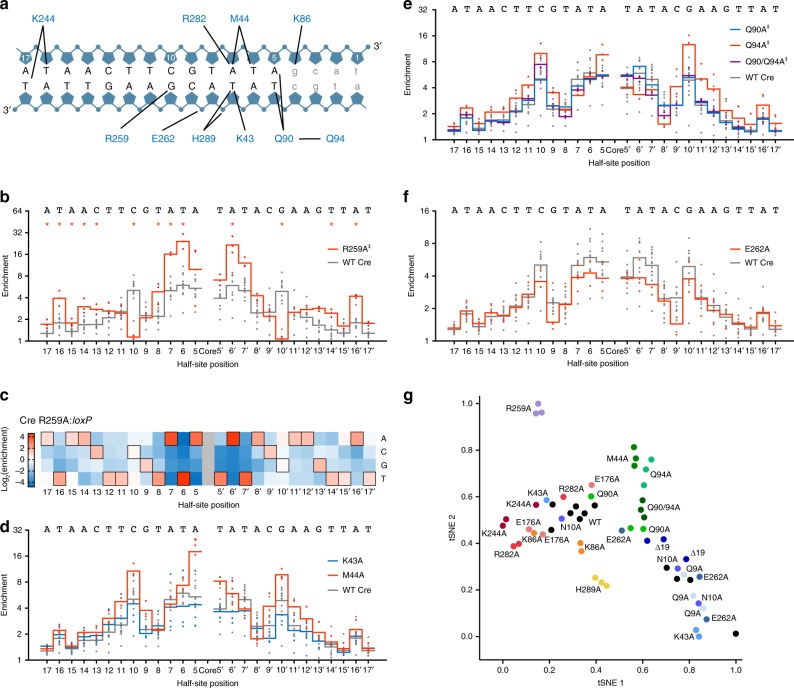
Determinants of Cre:*loxP* specificity identified by Rec-seq on wild-type Cre and Ala-substituted Cre variants. **a** Protein:DNA contacts for Cre:*loxP* inferred from crystal structures^8^. **b** Rec-seq enrichment profile for the CreR259A variant (red line) and wild-type Cre (gray line). **c** Heat map of Rec-seq enrichment values for the CreR259A variant showing the log_2_ of the enrichment value for each nucleotide at each position in *loxP* relative to the canonical base (black outline). **d**–**f** Rec-seq enrichment profiles for Ala-substituted Cre mutants (colored lines) and wild-type Cre (gray lines). For **b**–**f** values represent the geometric mean of *n* = 3 or *n* = 11 (wild-type Cre) independent replicates (dots) conducted at 37 °C for 30 min at a 1:3 protein:DNA ratio. **g** The results of Rec-seq experiments on wild-type Cre (black dots) and variants thereof (colored dots) visualized using t-SNE multi-dimensional proximity analysis^37^, showing that experimental replicates are clustered by similarity across all specificity features. Significant differences (*p* ≤ 0.05) relative to wild-type Cre at individual nucleotides (Student's t-test; colored asterisks) and across the full log-enrichment profile (Mann-Whitney U test; ‡) are indicated. Source data are provided as a Source Data file

The complexity of Cre:*loxP* interactions has challenged Cre engineering efforts^8,13,20^. To characterize these interactions, we constructed 14 Cre mutants with Ala substitutions at residues known to make contacts with *loxP* (Fig. 2a), purified each variant, and performed Rec-seq to map the functional relationship between specific residues and the DNA sequence preferences of Cre. Comparison of the Rec-seq profile of Cre mutants and wild-type Cre yielded novel insights into each residue’s contribution to DNA specificity across the entire *loxP* site.

Structural and mutagenesis studies^23,26,31^ suggested that mutation of Arg259 would affect specificity at half-site position 10. Indeed, the Arg259→Ala variant showed a drop in enrichment at position 10 (from 5.0-fold for wild-type Cre to 1.1-fold for the mutant), with a modest preference for C or T in the left half-site and G or A in the right half-site (Fig. 2b, c). The Arg259→Ala mutant also showed increased preference at virtually every other position in the *loxP* site, with especially high preferences at positions 5–7 and 16. This observation is consistent with an energetic tradeoff—as we proposed for zinc fingers, TALEs, and Cas9^32–34^—in which the loss of binding energy from Ala substitution at Arg259 (ref. ^23^) necessitates greater fidelity at other protein:DNA contacts to retain sufficient binding to support recombination, even when these interactions take place far from the altered residue. These long-range effects cannot be inferred from the Cre:*loxP* structure, highlighting the utility of unbiased, high-resolution specificity profiling.

Rec-seq also helped illuminate determinants of specificity at *loxP* positions 5–7, which are less well-understood than the determinants at position 10. Candidate interacting residues are distributed through three regions of Cre: helix B, helix D, and the loop between helices J and K (Fig. 2a). Rec-seq profiles of Ala mutants at potential interacting residues demonstrate differing impacts of neighboring residues. For example, in helix B, Rec-seq of the Lys43→Ala mutant resulted in a modest drop in specificity relative to wild-type Cre, while Met44→Ala resulted in higher preference at positions 5 and 10 (Fig. 2d). In helix D, the Lys86→Ala variant showed minimal differences from wild-type Cre (Supplementary Fig. 7a), while the Gln90→Ala variant showed overall lower enrichment (Fig. 2e). In the loop between helices J and K, the Arg282→Ala mutant showed higher, rather than lower, DNA specificity across *loxP* (Supplementary Fig. 7b). These results demonstrate that Cre’s apparent preference at positions 5–7 results from multiple weak or indirect interactions, rather than being strongly determined by residues proximal to these positions.

In addition, Rec-seq identified a contribution from a secondary residue previously unknown to participate in specifying positions 5–7. Ala substitution at Gln94 resulted in lower specificity at positions 6 and 7 but compensatory increases elsewhere (Fig. 2e), even though Gln94 does not directly contact the DNA, but instead engages in hydrogen bonds with Gln90^35^. Double Ala substitution at both Gln90 and Gln94 performed similarly to the Gln90→Ala single mutant (Fig. 2e), suggesting that the DNA-contacting residue Gln90 plays the dominant role in defining DNA specificity among the two residues. Together, Rec-seq profiling clarifies the many interactions that together define Cre recognition at positions 5–7, and highlights the important roles of secondary and indirect interactions.

We also applied Rec-seq to examine the role of Glu262, which forms backbone and nucleobase contacts at half-site position 9 (ref. ^31^). Gly or Ala substitutions at Glu262 were previously shown to increase tolerance for mismatches at non-contacted *loxP* positions (e.g., bases 11–12)^22^. The Rec-seq profile of the Glu262→Ala variant showed a drop in specificity at the proximal positions 8–9 (Fig. 2f), but also decreased specificity at positions 5–7 and 10, consistent with previous findings of Glu262’s role in enforcing substrate fidelity^31^.

Rec-seq revealed new roles for residues that were not previously known to play a long-range specificity-determining role, such as Lys244 and Glu176. Rec-seq of Lys244→Ala showed a decrease in specificity at the proximal position 17, but otherwise broadly increased specificity for *loxP* (Supplementary Fig. 7b). Glu176 is a highly conserved residue among tyrosine recombinases that is proximal to the Cre active site, not the DNA substrate^28^, but Rec-seq of Glu176→Ala showed broadly increased specificity (Supplementary Fig. 7c). In addition, Rec-seq illuminates contradictory observations about the role of the Cre N-terminus in DNA specificity. While the N-terminus is unresolved in crystal structures and can be truncated with no apparent effect^36^, laboratory evolution of Cre yielded mutations at Gln9 and Asn10 that are essential for evolved activity^35^. Rec-seq profiles of Δ19 Cre (lacking the first 19 amino acids), Gln9→Ala, and Asn10→Ala each showed no significant differences compared to wild-type Cre (Supplementary Fig. 8). These results suggest that while individual residues in the N-terminus may participate in catalysis, they are unlikely to contribute substantially to *loxP* recognition. Collectively, these findings highlight the ability of Rec-seq to reveal specificity determinants regardless of the proximity between the contributing residue and the DNA base being influenced.

Our understanding of SSR:DNA interactions largely arises from static crystal structures. While structures provide a list of possible interactions based on proximity, Rec-seq generates a functional map of residues that contribute to specificity. To visually represent one such map, we used the t-SNE algorithm^37^ to correlate the results of individual Rec-seq experiments using multi-dimensional similarity analysis (Fig. 2g). The proximity of experiments in the t-SNE visualization relates their similarity across the full Rec-seq profile. For example, the cluster containing Met44 and Gln94 represents the functionally similar residues contributing to specificity at positions 5–7, while other residues proximal to the same bases (Lys43, Lys86, Arg282) appear separately, consistent with their differing roles. Replicates of Rec-seq experiments with wild-type Cre cluster together toward the middle of the graph; Ala-substituted mutants that increase sequence preference appear to the left of the wild-type grouping, while preference-diminishing variants cluster to the right. By revealing and correlating the individual roles of residues in determining DNA recognition across the entire substrate site at single-nucleotide resolution, Rec-seq greatly enhances our understanding of SSR:DNA interactions.

### Rec-seq of evolved Cre variants

**Fig. 3:**
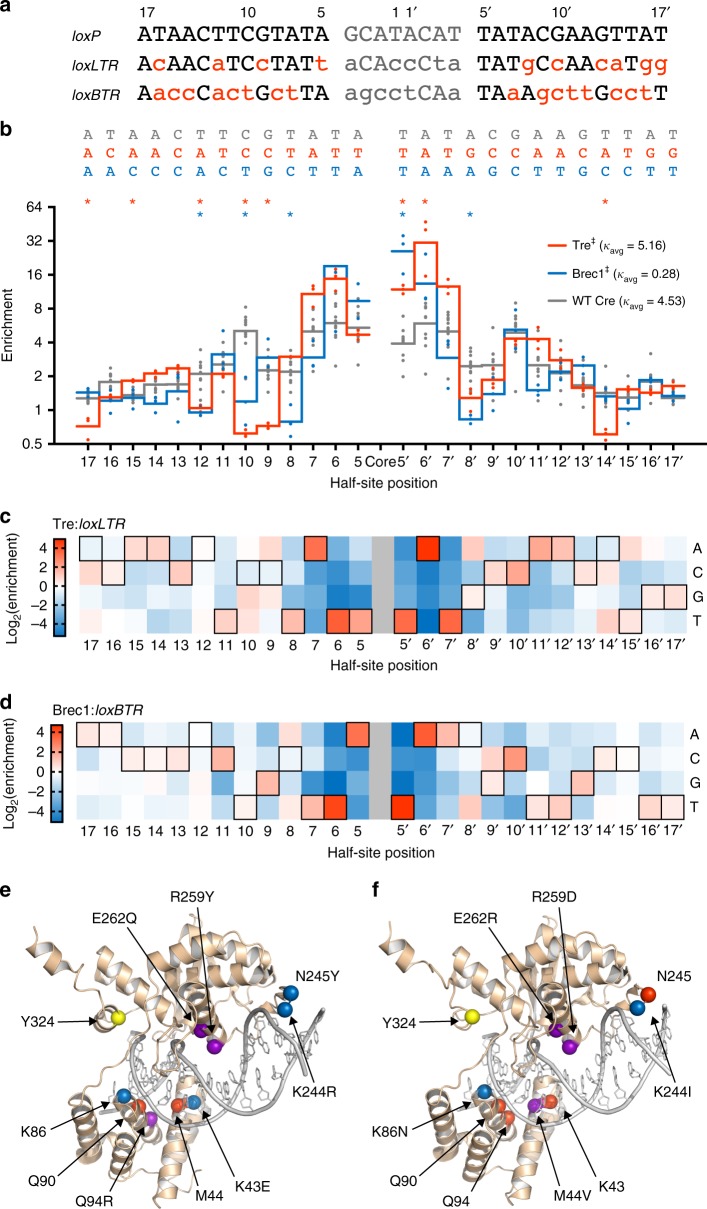
DNA specificity of evolved Cre variants revealed by Rec-seq. **a** DNA sequences of *loxP*, *loxLTR*, and *loxBTR* showing differences relative to *loxP* (red). **b** Rec-seq specificity profiles for Tre, Brec1, and wild-type Cre. Values represent the geometric mean of *n* = 3 or *n* = 11 (wild-type Cre) independent replicates (dots) conducted at 37 °C for 30 min at a 3:1 protein:DNA ratio. Significant differences (*p* ≤ 0.05) relative to wild-type Cre at individual nucleotides (Student's t-test; colored asterisks) and across the full log-enrichment profile (Mann Whitney U test; ‡) are indicated. **c**, **d** Heat map of Rec-seq enrichment values for Tre and Brec1 showing the log_2_ of the enrichment value for each nucleotide at each position in *loxLTR* or *loxBTR* relative to the target base (black outline). **e**, **f** Specifying interactions mapped onto the structure of Tre in complex with *loxLTR*^35^ (**e**) or Brec1 interactions mapped onto the structure of Cre in complex with *loxP*^65^ (**f**). The catalytic Tyr (yellow), residues with conserved interactions at unchanged positions relative to *loxP* (red), residues proximal to positions of decreased specificity (blue), and residues that participate in recognition of the new target site (purple) are depicted as spheres. One-letter amino acid labels indicate the Cre residue at that position and the identity of the mutation in Tre or Brec1, if any. Source data are provided as a Source Data file

We next sought to interrogate the basis of specificity for laboratory-evolved Cre variants, which have never been characterized comprehensively. We first applied Rec-seq to Tre, which was evolved to recognize *loxLTR*, a sequence that differs from *loxP* at 50% of base pairs^14^ (Fig. 3a). Rec-seq revealed that Tre showed relaxed specificity relative to Cre at multiple positions in *loxLTR*, including positions 9, 10, 12, and 17 in the left half-site and position 14 in the right half-site (Fig. 3b, c). Tre showed concomitant increased substrate nucleotide preference at positions 5–7, providing further support for an energetic tradeoff model described above. Some of this heightened specificity in Tre occurred at base pairs that were unchanged between *loxP* and *loxLTR* (5 of 6 base pairs among positions 5–7 in both half-sites). In addition, Tre maintained enhanced sequence preference at left half-site position 5 and right half-site position 10, which both differ between *loxLTR* and *loxP*. This finding is consistent with the Tre:*loxLTR* co-crystal structure^35^, which predicts hydrogen bonding interactions between Gln90 and Arg94 side chains in Tre and the T•A base pair at position 5 (Fig. 3e). Preferences at these altered positions are consistent with evolved recognition for the *loxLTR* substrate, and are likely necessary to offset the loss of DNA interactions at other positions.

We also applied Rec-seq to Brec1, a Cre variant evolved to recognize the *loxBTR* target, which differs from *loxP* at 68% of base pairs^15^ (Fig. 3a). Similar to Tre, the Rec-seq profile of Brec1 showed evidence of tradeoffs between loss of protein:DNA interactions at some positions within the half-site and enhanced specificity for critical positions elsewhere. Brec1 showed diminished preference at position 8 in both half-sites and positions 10 and 12 in the left half-site, and conserved specificity for positions 5 and 6 in both half-sites of *loxBTR* (Fig. 3b, d). Additionally, Brec1 maintained enhanced specificity for right half-site position 10, which differs between *loxP* and *loxBTR*. These regions of high specificity likely represent a mixture of conserved and novel Brec1:*loxBTR* interactions (Fig. 3f), the presence of which may be required to offset the loss of binding interactions in other regions of the target site.

For both evolved variants, Rec-seq revealed that target recognition arose from a combination of conserved interactions, evolved recognition at important half-site positions, and relaxed specificity. Our results support the findings from structural characterization of Tre:*loxLTR*, and also suggest the presence of novel interactions between Brec1 and *loxBTR*, which have not yet been co-crystallized.

### Rec-seq of Dre, VCre, and Bxb1 recombinases

**Fig. 4:**
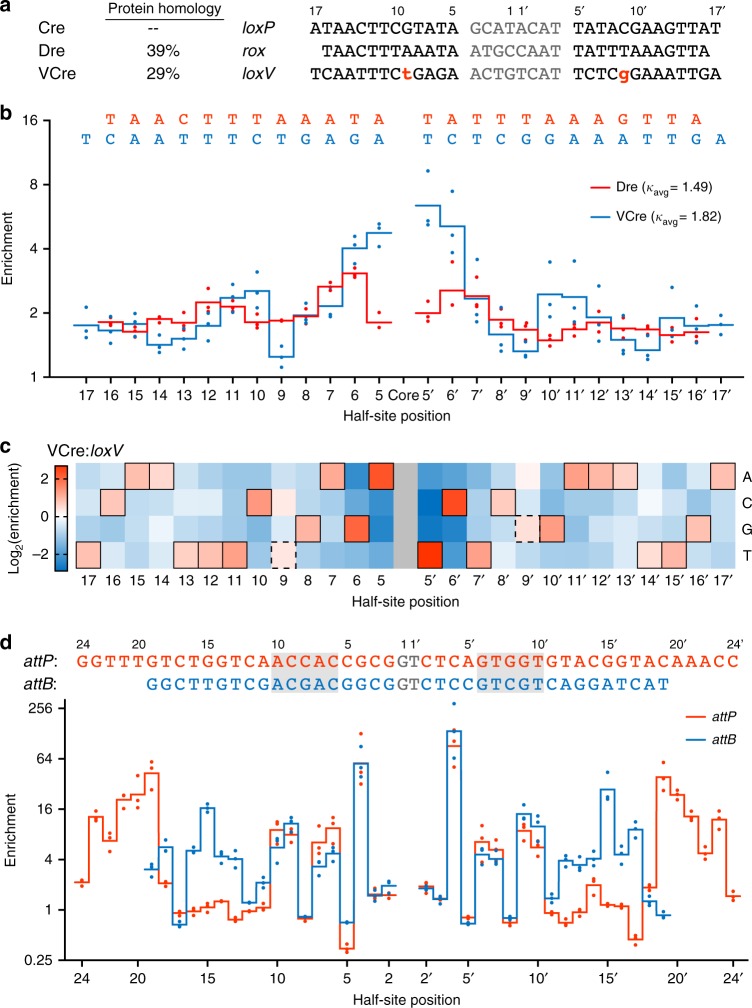
Rec-seq profiles of Dre, VCre, and Bxb1 site-specific recombinases. **a** Cre, Dre, and VCre differ at the protein sequence level, and bind different recognition targets. **b** Rec-seq of tyrosine recombinases Dre and VCre. Values represent the geometric mean of *n* = 3 independent replicates (dots) conducted at 37 °C for 30 min at a 3:1 protein:DNA ratio. **c** Heat map of Rec-seq enrichment values for VCre showing the log_2_ of the enrichment value for each nucleotide at each position in *loxV* relative to the canonical base (black outline). **d** Rec-seq of serine integrase Bxb1 on its substrates *attP* and *attB*. Both substrates contain a conserved ACNAC motif (gray box). Values represent the geometric mean of *n* = 3 independent replicates (dots) conducted at 37 °C for 30 min at a 3:1 protein:DNA ratio. Source data are provided as a Source Data file

Next, we applied Rec-seq to non-Cre recombinases, most of which remain unexplored as genome editing agents. We performed Rec-seq on Cre relatives Dre^38^ and VCre^39^ using half-site libraries based on their target substrates *rox* and *loxV*, which differ from *loxP* at 25% and 46% of non-core positions, respectively (Fig. 4a). Dre and VCre preferred the canonical base at nearly every position in their target sites, similar to wild-type Cre (Figs. 4b, 1c). Though their canonical sequences were enriched in Rec-seq, Dre and VCre profiles revealed several half-site positions with heightened preference relative to neighboring positions. Dre showed the strongest preference for half-site positions 6, 7, and 12, while VCre enriched most strongly at positions 5, 6, 10, and 11 (Fig. 4b). Additionally, VCre showed a unique preference at position 9, which is asymmetric in *loxV* (Fig. 4a). We observed binary recognition at position 9: T or a C is preferred in the left half-site, with G or A preferred in the right half-site (Fig. 4c). We hypothesize that these enrichment profile features result from direct interactions between Dre:*rox* and VCre:*loxV*, which may be confirmed by crystallization or in-depth characterization of Dre and VCre.

We also applied Rec-seq to the serine integrase Bxb1^40^, which performs strand exchange between two different DNA substrates^1^, *attP* and *attB* (Fig. 4d). Rec-seq with libraries derived from both substrates revealed that Bxb1 maintains two partially overlapping recognition modes to distinguish and selectively recombine two targets that are distinct in sequence and length. We hypothesized that Bxb1 would show strongest enrichment levels at regions of homology between *attP* and *attB*. Both sites contain a G•C base pair at position 4 and 4′, and, in agreement with the literature^41^, we observed nearly absolute specificity for these positions in both substrates (Fig. 4d). Rec-seq profiles also showed enrichment of the ACNAC motif present at positions 6–10 in both the *attP* and *attB* half-sites (Fig. 4d), consistent with the presence of specifying protein:DNA interactions operating on both targets.

Outside of these regions of homology, Bxb1 showed divergent recognition patterns for each substrate. In Rec-seq experiments with *attP* substrates, Bxb1 enriched strongly at half-site positions 19–23 (Fig. 4d). Enrichment at these positions is consistent with previous reports of a preference for distal bases within *attP* for Bxb1 (ref. ^41^) and other integrases^42^. This enrichment largely occurs at positions outside the *attB* minimal site, which consists of two 19-bp half-sites^40^. Bxb1 showed the strongest preference for positions 13–16 in both half-sites of *attB*, but minimal preference for the same region in *attP* (Fig. 4d). These findings collectively support a model^42^ in which Bxb1 enforces fidelity of two asymmetric substrates by adopting overlapping but distinct recognition modes for *attP* and *attB*.

Together, the application of Rec-seq to the characterization of non-Cre recombinases lends support to our model of SSR substrate preferences, uncovers previously unreported specificity determinants, and demonstrates the broad applicability of the Rec-seq method.

### Off-target recombinase activity predicted by Rec-seq

Finally, we investigated the ability of Rec-seq to predict off-target activity of SSRs. Before candidate genome editing agents can be used for therapeutic applications, their potential for off-target activity must be assessed^43^. Broadened substrate tolerance is anticipated for laboratory-evolved recombinases, as proteins undergoing evolution commonly acquire substrate promiscuity before gaining specificity for the new target^44^. Indeed, we observed relaxed specificity at multiple positions in the Rec-seq profiles of evolved Cre variants Tre and Brec1 (Fig. 3b). We used Rec-seq data to predict potential off-target substrates for Tre and Brec1 and then assayed the ability of these evolved recombinases to process predicted substrates, including mismatched “synthetic” substrates enriched from Rec-seq libraries, as well as pseudosites present in the human genome.

To generate candidate off-target substrates for Tre and Brec1, we first identified non-target half-site sequences that appeared with high abundance in the post-recombinase-treated dataset. For each evolved SSR, we chose four left and right half-site sequences, L1–L4 and R1–R4, that contained two or three mutations at various half-site positions. The mismatched sequences were observed at 2.7- to 18-fold higher abundance after recombinase treatment versus the input library abundance, compared to the matched *loxLTR* and *loxBTR* sequences, which were enriched 3.0- and 3.4-fold, respectively (Supplementary Tables 2 and 3).

**Fig. 5:**
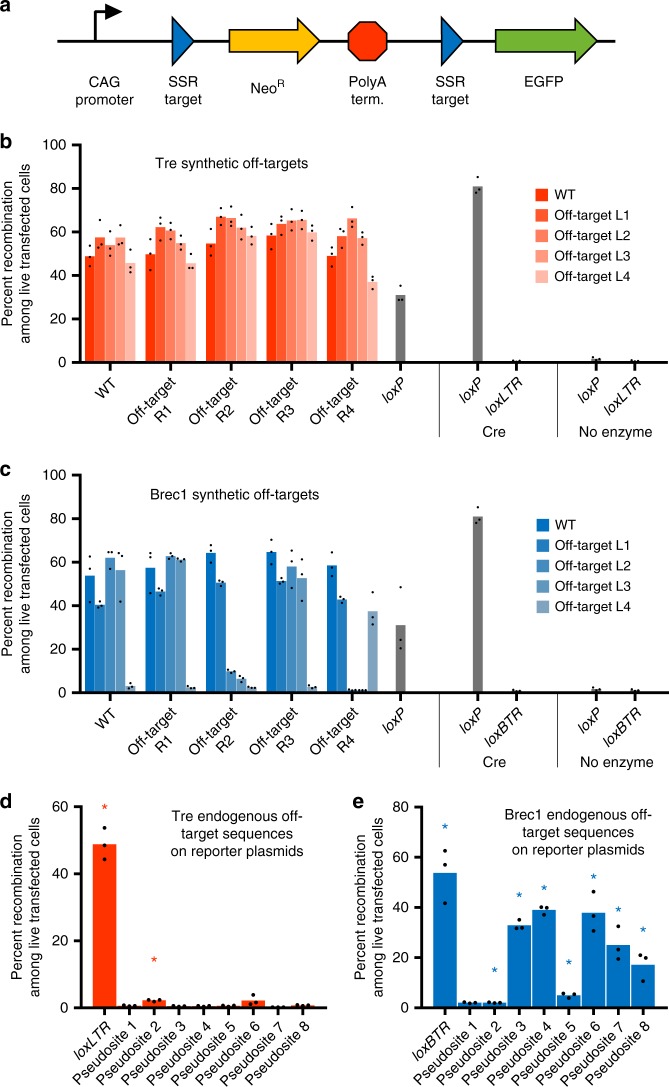
Off-target recombinase activity predicted by Rec-seq. **a** Cells were transfected with recombinase expression plasmid and an EGFP reporter plasmid containing candidate recombinase substrates flanking a poly-A terminator that blocks *EGFP* transcription. Tre and Brec1 activity on synthetic off-target substrates (**b**, **c**) and predicted endogenous human genomic pseudosites (**d**, **e**) was measured as the fraction of cells exhibiting EGFP fluorescence. The percentage of EGFP-positive cells shown is of transfected cells (determined by gating for the presence of co-transfected plasmid constitutively expressing mCherry) and 10,000 live events were recorded for each experiment. Data are represented as the mean (bars) of three independent biological replicates (dots). For **d**, **e**, significant differences (*p* ≤ 0.05) relative to no-enzyme control samples are indicated (Student's two-tailed t-test; colored asterisks). Source data are provided as a Source Data file

We assessed the activity of Tre and Brec1 on these synthetic substrates in human cells using a reporter plasmid containing pairwise combinations of L1–L4 and R1–R4 half-sites flanking a poly-A terminator that blocks *EGFP* transcription (Fig. 5a). In this reporter system, recombinase-mediated deletion of the terminator restores *EGFP* expression. We co-transfected HEK293T cells with the reporter plasmid and a plasmid expressing either Tre or Brec1, then used the fraction of cells exhibiting EGFP fluorescence to assess the activity on each target. Both Tre and Brec1 showed comparable or higher activity on the majority of tested synthetic targets relative to their cognate substrate (Fig. 5b, c), even though these substrates contained up to six mismatches. These findings are consistent with relaxed specificities of the evolved variants observed in Rec-seq, and suggest that in vitro substrate preferences of SSRs revealed by Rec-seq are predictive of the activity in a reporter plasmid in human cells.

We also assessed whether Rec-seq data alone could predict the activity of Tre and Brec1 on endogenous human genomic sequences. To identify potential pseudosites, we searched the human genome for sequences that contained the Tre or Brec1 minimal substrate motif, inferred from positions within each half-site with Rec-seq enrichment values greater than 2. Using the RSAT motif scanner^45^ and search parameters A_14_C_13_NT_11_NNT_8_A_7_T_6_T_5_NNNNNNNNT_5′_A_6′_T_7′_NNC_10′_A_11′_A_12′_ for Tre and C_11_NG_9_NT_7_T_6_A_5_NNNNNNNNT_5′_A_6′_A_7′_NNC_10′_NT_12′_G_13′_ for Brec1, we identified eight human genomic off-target substrates per SSR, each containing 6–11 non-core mismatches (Supplementary Tables 4 and 5). These candidate pseudosites were cloned into the EGFP reporter, and Tre and Brec1 activity was assessed in HEK293T cells as described above. Tre showed significant activity on one of eight endogenous pseudosites (Fig. 5d). Brec1, however, showed robust activity (>15%) on five of eight endogenous pseudosites, with significant activity on seven (Fig. 5e). We confirmed previously reported activity of Brec1 on singly mismatched substrates (Supplementary Fig. 9; Supplementary Table 6). We also observed Brec1 activity in human cells on human genomic off-target sequences that were previously identified solely on the basis of *loxBTR* sequence similarity, and found to not undergo recombination by Brec1 in bacterial assays^15^ (Supplementary Fig. 9; Supplementary Table 6). We attribute this discrepancy, as well as our finding of substantial Tre and Brec1 activity on *loxP*, to differences in SSR performance in mammalian cells compared to the *Escherichia coli*-based assays. These findings suggest that Rec-seq can predict the activity of SSRs on off-target loci including endogenous human genomic pseudosites using only in vitro enrichment data.

## Discussion

Rec-seq is a powerful, high-throughput sequencing-based method that reveals the DNA sequence preferences of SSRs, including specificity determinants not evident from structural studies. We validated Rec-seq with Cre:*loxP*, and used it to characterize the specificity contributions of over a dozen Cre residues. Rec-seq profile results support a model for recombinase specificity in which productive recombination requires sufficient binding energy, and loss of one protein:DNA interaction necessitates compensatory increases in fidelity at other (often distant) regions of *loxP*. We also used Rec-seq to accurately predict off-target activity of potential therapeutic recombinases Tre and Brec1. Our findings corroborate previous biochemical and structural characterization of recombinases and reveal numerous unanticipated insights about Cre and other SSRs, including asymmetric substrate preferences of Cre and long-range interactions of unexpected residues.

Rec-seq represents a major improvement over previous approaches to characterizing the specificity of SSRs, which typically require assaying recombinase activity on each substrate of interest in isolation^41,46–48^. Such experiments are labor-intensive, making it impractical to test even all doubly mutated substrates, and do not interrogate the relative preference for multiple competing substrates. More sophisticated methods involve generating a pool of randomized substrates with degenerate primers^49–51^ or sheared genomic DNA^52^, but these methods use bacterial antibiotic selection to isolate recombinase substrates, and the resolution of such profiling methods is therefore limited by the need to DNA sequence many individual colonies.

In contrast, Rec-seq is an unbiased and rapid method for characterizing SSR substrate preferences at high resolution. The experiments are simple and inexpensive, require no specialized training or equipment, and are easily parallelized. Multiple Rec-seq experiments can be conducted by one researcher in a single day beginning with purified protein and synthesized DNA. We demonstrate the generality of Rec-seq by characterizing not only a widely studied recombinase, Cre, but also distantly related tyrosine SSRs with limited biochemical characterization, as well as an unrelated serine integrase.

Rec-seq also enables experimentally driven off-target substrate prediction for recombinases. The predictive ability of computational searches for recombinase pseudosites in a genome of interest^53,54^ is limited by the extent of knowledge about recombinase substrate preferences. Empirical methods for detecting SSR pseudosites include overexpressing the recombinase in mammalian cells and identifying sites of genomic modification^55,56^. Rec-seq increases the predictive ability of these methods by generating high-resolution, nucleotide-level DNA specificity profiles of recombinases from libraries of DNA sequences that are orders of magnitude larger than the size of typical mammalian genomes, and that contain a much larger fraction of sequences related to cognate DNA substrates. We used these features of Rec-seq to accurately anticipate Tre and Brec1 activity on pseudosites present in the human genome.

Despite these significant advantages, Rec-seq has its own limitations. In its current form, Rec-seq is incompatible with recombinases that require supercoiled substrates^1^ due to the linear oligonucleotide origins of the substrate variants. Rec-seq also requires that the researcher can generate purified recombinase and can identify conditions that support in vitro activity on Rec-seq library substrates. Finally, Rec-seq results are derived from experiments in which only one half-site (L1 or R2) contains mutations while the other three half-sites contain the wild-type sequence, preventing Rec-seq from revealing specificity changes that only arise when multiple changes in different half-sites are simultaneously present.

Rec-seq may facilitate the development of therapeutic recombinases with tailor-made specificities. Generating Rec-seq profiles of different SSRs would increase the pool of potential starting points for retargeting SSRs. Thousands of SSRs are predicted to be encoded in sequenced genomes^57,58^, and their Rec-seq profiling would require only knowledge of a cognate substrate sequence and in vitro conditions that support SSR activity. Broad profiling of diverse SSRs may also uncover family members with desirable traits as genome editing agents, such as the binary specificity of VCre for the asymmetric position 9 in *loxV* and dual substrate recognition by Bxb1 we observed in this study.

Rec-seq findings show that long-distance compensatory interactions play an underappreciated role in substrate recognition compared to the limited number of direct Cre:*loxP* contacts. Indeed, among all examined residues predicted to make direct protein:DNA contacts, Ala substitution at only one position (at Arg259) resulted in a near-complete loss of specificity for the proximal base. We also observed that extensive laboratory evolution of Tre and Brec1 resulted in few newly evolved interactions. Together, these findings and previous reports suggest that the dominant mode of substrate recognition for SSRs is not direct protein:DNA interactions, but instead a combination of multiple weak interactions and shape- and charge-complementarity.

## Methods

### General methods

All oligonucleotides and gBlocks were purchased from Integrated DNA Technologies (IDT). All enzymes and buffers were purchased from New England Biolabs (NEB) unless noted. PCR was performed using either Phusion U Green Multiplex PCR Master Mix (ThermoFisher Scientific) or Q5 Hot Start High-Fidelity 2x Master Mix (NEB). All plasmids were generated by USER cloning and transformed into One Shot Mach1 T1 *E. coli* (ThermoFisher Scientific) unless otherwise noted. Plasmids for mammalian cell transfection were prepared using an endotoxin-removal plasmid-purification system, ZymoPURE Plasmid Midiprep (Zymo Research Corporation).

### Cloning and purification of Cre and SSR variants

Ala-substituted Cre variants were generated by single-amplicon blunt-end ligation cloning of 5′-phosphorylated PCR products generated from a previously described pET-His-Cre vector^59^. Expression vectors for other proteins were generated by USER cloning using gBlocks (Tre, VCre) or previously described plasmids (Dre^60^, Bxb1^61^) as a PCR template. Protein sequences and primers used are listed in Supplementary Notes 3 and 4, and plasmids for expression of Cre, Tre, Dre, VCre, and Bxb1 are available from Addgene.

BL21-Star (DE3)-competent *E. coli* cells were transformed with plasmids encoding Cre or other recombinases with a His purification tag. A single colony was grown overnight in 2× YT broth containing 50 µg/mL carbenicillin at 37 °C. The cells were diluted 1:250 into 250 mL of the same media and grown at 37 °C until OD_600_ = 0.60. The cultures were incubated on ice for 20 min and protein expression was induced with 1 mM isopropyl-β-d-1-thiogalactopyranoside (IPTG; GoldBio). Expression was sustained for 14–16 h with shaking at 16 °C. The subsequent purification steps were carried out at 4 °C. Cells were collected by centrifugation at 8000*g* for 20 min and resuspended in cell-collection buffer (100 mM tris(hydroxymethyl)-aminomethane (Tris)-HCl, pH 8.0, 1 M NaCl, 20% glycerol, 5 mM tris(2-carboxyethyl)phosphine (TCEP; GoldBio), and 1 complete EDTA-free protease inhibitor pellet (Roche) per 120 mL buffer used). Cells were lysed by sonication (4 min total, alternating 1 s on and 1 s off) and the lysate cleared by centrifugation at 12,000*g* (20 min).

The cleared lysate was incubated with His-Pur nickel nitriloacetic acid (nickel-NTA) resin (4 mL resin per liter of culture; ThermoFisher) with rotation at 4 °C for 60–90 min. The resin was washed with 50 mL of cell-collection buffer before bound protein was eluted with elution buffer (100 mM Tris-HCl, pH 8.0, 0.5 M NaCl, 20% glycerol, 5 mM TCEP, 500 mM imidazole). The resulting protein fraction was injected into a Slide-A-Lyzer dialysis cassette (10-kDa molecular-weight cutoff; ThermoFisher) and dialyzed for 14–16 h at 4 °C in approximately 100-fold excess storage buffer (100 mM Tris-HCl, pH 8.0, 20% glycerol, 5 mM TCEP). The dialyzed protein fraction was then concentrated using a column with a 10-kDa cutoff (Millipore) centrifuged at 3000 *g*. Proteins were quantified with Reducing Agent Compatible Bicinchoninic acid (BCA) assay (Pierce Biotechnology), snap-frozen in liquid nitrogen, and stored in aliquots at −80 °C.

Brec1 protein was provided by Dr. Gretchen Meinke and Professor Andrew Bohm, Tufts University School of Medicine. The protein contained a Leu163Phe stabilizing mutation and an N-terminal TEV-cleavable His-tag.

### In vitro extension of library oligonucleotides

DNA oligonucleotides containing the recombinase target sequence and a 3′ hairpin were diluted to 1 µM in nuclease-free water (GE Life Sciences) and NEBuffer 2 in a total volume of 25 µL. The oligonucleotides were heated to 95 °C and slow-cooled to 37 °C to anneal the hairpin, before adding 10 nmol dNTP solution mix and 5 units of Klenow Fragment (3′→5′ exo-) polymerase and incubating for 60–90 min. The extension reaction was stopped by incubation at 75 °C for 20 min, and extended DNA was stored at 4 °C for up to 1 week. Sequence information for the DNA oligonucleotides can be found in Supplementary Note 2, and a sample protocol is included in the Supplementary Methods.

### In vitro recombination assays

Each recombination reaction contained one left-hairpin and one right-hairpin substrate oligonucleotides with only one randomized half-site per reaction. In a total reaction volume of 50 µL, recombinase (0.66 pmol for a 1:3 ratio of protein:DNA) was mixed with 1 pmol per oligonucleotide in nuclease-free water and Cre Recombinase Buffer (NEB) for 30 min at 37 °C. Addition of PB buffer (Qiagen, 200 µL) stopped the reaction, and DNA was purified with Minelute columns (Qiagen). The purified DNA was digested with the addition of NEBuffer 4, 1 mM adenosine 5′-triphosphate (ATP), and exonucleases I (20 units), III (100 units), and V (10 units) and incubated for 45–90 min at 37 °C. The reactions were purified with Minelute columns and the remaining DNA was amplified to the middle of linear range by qPCR (1 µL input DNA, 25 µL reaction volume) using iTaq polymerase (Universal SYBR Green Supermix; Bio Rad) and primers listed in Supplementary Note 4. PCR conditions were as follows: 98 °C, then repeated cycles of 98, 57, and 72 °C extension for 5 s. Quantitative PCR was used to ensure that the library composition was not affected by PCR bias and that the recombinase-treated samples were more abundant than a no-recombinase negative-control sample. Amplified DNA was purified using Minelute columns and barcoded with a second round of qPCR (0.5 µL input DNA) before being prepared for sequencing on an Illumina MiSeq as described below.

The above protocol was modified to reflect empirical differences in the optimal reaction conditions for assays with evolved Cre variants and unrelated SSR family members. The recombination reactions with Tre, Brec1, Dre, VCre, and Bxb1 were carried out with a five-fold increase in concentration of both enzyme and substrate DNA. For Tre and Brec1, recombination buffer was supplemented with 100 ng BSA. For Dre and VCre, reactions were supplemented with 100 ng BSA and 1 mM dithiothreitol (DTT). For Bxb1, reactions were carried out in Bxb1 reaction buffer^62^ (20 mM Tris-HCl, pH 7.5, 10 mM EDTA, 25 mM NaCl, 10 mM spermidine, and 1 mM DTT) supplemented with 100 ng BSA. All reactions were carried out at 3:1 protein:DNA ratios for 30 min at 37 °C.

### Sequencing and analysis of DNA amplicons

The unique forward and reverse primers used in the first-round PCR contained a constant region 5′ to the annealing region (forward: 5′-ACACTCTTTCCCTACACGACGCTCTTCCGATCTNNNN-3′, reverse: 5′-TGGAGTTCAGACGTGTGCTCTTCCGATCT-3′), which facilitated binding of barcoding primers to amplified DNA for a second-round PCR.

The second-round PCR used primers with three regions: a 5′ constant region allowing the amplicon to bind to the Illumina flow cell (italicized), an 8-base barcoding region (X), and a 3′ constant region allowing the barcoding primer to bind to the first-round PCR amplicon (in bold). Examples of primer sequences are:

forward: 5′-*AATGATACGGCGACCACCGAGATCTACAC*XXXXXXXX**ACACTCTTTCCCTACACGAC-3′,**

reverse: 5′-CAAGCAGAAGACGGCATACGAGATXXXXXXX**GTGACTGGAGTTCAGACGTGTGCTCTTC-3′**

Sequencing adapters and dual-barcoding sequences are based on the TruSeq Indexing Adapters (Illumina). Barcoded samples were quantified using the Qubit dsDNA HS Kit (ThermoFisher) according to the manufacturer’s instructions. Sequencing of pooled samples was performed using a single-end reads of 225–250 bases on the MiSeq (Illumina) according to the manufacturer’s instructions.

### Rec-seq data analysis

Sequencing reads were automatically demultiplexed using MiSeq Reporter (Illumina) and Fastq files were analyzed using custom software tools written in Python 3, made available online at https://github.com/broadinstitute/rec-seq. In brief, post-recombination sequencing reads that contained the matched target core sequence were aligned to the native target sequence, with no gaps allowed. After alignment, reads with excessive numbers of mismatches were determined to be the result of sequencing errors, e.g., reads containing indels. Therefore, aligned reads with greater than six mismatches relative to the reference sequence were filtered out of subsequent analysis. For the remaining sequences, at each position in the recombinase target, the abundance of the canonical base (*A*_*i*_) and the sum of the non-canonical bases (*B*_*i*_) were calculated. The same analysis was performed for the sequencing reads of the input library, but the abundances of the canonical base and the non-canonical bases were expressed as fractions *α*_*i*_ and *β*_*i*_. The enrichment score for each position was then calculated as the ratio *r*_*i*_ = (*A*_*i*_/*B*_*i*_)/(*α*_*i*_/*β*_*i*_) (see Supplementary Note 1 for enrichment score derivation). Analysis was performed separately for the left and right half-sites, using as input the sequencing reads from experiments with either L1- or R2-randomized half-sites (see Fig. 1b).

Significance of log-enrichment values was calculated by performing the Student’s *t*-test assuming equal variance for each individual position of each SSR variant relative to wild-type Cre, and the effect of multiple comparisons was counteracted using the Bonferroni correction. A paired *t*-test was used to compare the asymmetry between the left and right half-site log-enrichment values for wild-type Cre (Supplementary Fig. 6a). We calculated the significance of differences along the full substrate log-enrichment profile using the two-sided Mann–Whitney *U* test. To do so, we compared the absolute value of the residuals for wild-type Cre and each enzyme variant, and applied the Bonferroni correction. Significance values can be found in Supplementary Tables 7–9.

### Cloning of mammalian expression and reporter plasmids

Mammalian expression plasmids were constructed via the ligase cycling reaction (LCR) method^63^ using a pCMV vector and gBlocks encoding Tre and Brec1. Briefly, 5' phosphorylated primers were used to amplify the pCMV vector and the recombinase insert, and assemblies were conducted using single-stranded bridging oligonucleotides which spanned the cloning junctions and the optimized LCR protocol. Primers used are listed in Supplementary Note 4.

The pCALNL-GFP subcloning vector, pCALNL-EGFP-BsaI, was used to clone all reporter plasmids and was based on the previously described pCALNL-EGFP-Esp3I vector^19^. The *Bsa*I site in the ampicillin gene of the pCALNL-EGFP-Esp3I vector was first removed by Gibson assembly of BsaI-HFv2-digested plasmid and a dsDNA oligonucleotide with Gibson overhangs and a point mutation ablating the *Bsa*I site. The pCALNL-EGFP-BsaI plasmid was created by Golden Gate assembly with the modified pCALNL-EGFP-Esp3I vector and a PCR product bearing a pTET-mRFP cassette flanked by *Bsa*I and *Esp*3I sites. Golden Gate reactions were set up and performed as described previously with *Esp*3I (ThermoFisher Scientific)^64^. The donor vector, containing the neomycin-terminator cassette, was constructed by USER cloning using a PCR product of the cassette from pCALNL-EGFP-Esp3I and a pUC-Kan vector.

pCALNL-EGFP *loxP*, *loxLTR*, and *loxBTR* reporter plasmids were created by Golden Gate assembly with the pCALNL-EGFP-BsaI acceptor vector, pBT100-neomycin-terminator donor vector, and pairs of dsDNA oligonucleotides bearing recombinase target sites flanked by BsaI overhangs. Golden Gate reactions contained 0.1–1 pmol of each component, BsaI-HFv2 (20 units; NEB), and T4 DNA Ligase (20 units).

Plasmids for mammalian expression of Cre, Tre, and Brec1, as well as Golden Gate acceptor pCALNL-EGFP-BsaI and donor pBT100-neomycin-terminator are available from Addgene.

### HEK293T transfection and flow cytometry

HEK293T cells (ATCC CLR-3216) were cultured in Dulbecco’s modified Eagle’s medium (DMEM; Corning) supplemented with 10% fetal bovine serum (Life Technologies). Cells were seeded into 48-well poly-d-Lysine-coated plates (Corning) in the absence of antibiotics. Twelve to 15 h after plating, cells were transfected with 1 µL of Lipofectamine 2000 (ThermoFisher Scientific) using 250 ng of recombinase plasmid, 25 ng of reporter, and 10 ng of fluorescent protein expression plasmid as a transfection control. Cells were cultured for 3 days before they were washed with PBS (ThermoFisher Scientific) and detached from plates by the addition of TrypLE Express (ThermoFisher Scientific). Cells were diluted in 250 µL culture media and run on a BD LSR II analyzer. Exemplary flow cytometry plots are shown in Supplementary Fig. 10. Significance of recombinase activity measurements relative to no-recombinase control transfections was calculated by performing the Student’s two-tailed *t*-test assuming unequal variance.

### Reporting summary

Further information on research design is available in the Nature Research Reporting Summary linked to this article.

## Supplementary information

Supplementary Information

Reporting Summary

Source Data

## Data Availability

High-throughput sequencing data have been deposited in the NCBI Sequence Read Archive database under accession number PRJNA517947 (SRP182963). The source data underlying Figs. 1c, d, 2b–g, 3b–d, 4b–d, 5b–d, and Supplementary Figs. 1–4 and 6–9 are provided as a Source Data file. Other data are available upon request.
