# Addendum: High-resolution specificity profiling and off-target prediction for site-specific DNA recombinases

**DOI:** 10.1038/s41467-019-10892-9

**Published:** 2019-07-02

**Authors:** Jeffrey L. Bessen, Lena K. Afeyan, Vlado Dančík, Luke W. Koblan, David B. Thompson, Chas Leichner, Paul A. Clemons, David R. Liu

**Affiliations:** 1 Merkin Institute of Transformative Technologies in Healthcare, Broad Institute of Harvard and MIT, Cambridge, MA 02142 USA; 2 Department of Chemistry and Chemical Biology, Harvard University, Cambridge, MA 02138 USA; 3 Howard Hughes Medical Institute, Harvard University, Cambridge, MA 02138 USA; 4 Chemical Biology and Therapeutics Science Program, Broad Institute of Harvard and MIT, Cambridge, MA 02142 USA; 5 Google Inc., Mountain View, CA 94043 USA

**Keywords:** Genetic engineering, Gene targeting, Sequencing

Addendum to: *Nature Communications* 10.1038/s41467-019-09987-0, published online 26 April 2019.

The authors have become aware that ‘BTR pseudo-site 2’ inadvertently contains two incorrect nucleotides and does not match the sequence of the pseudo-site in the human genome. The data points corresponding to this construct in Fig. 5e and Supplementary Table 5 are therefore no longer valid. However, the overall conclusion that Rec-seq can predict the activity of site-specific recombinases on endogenous human genomic pseudo-sites of remains unaffected by this error. Updated versions of Fig. 5 and Supplementary Table 5 are presented below as Figures 1 and Table 1 respectively.

**Figure 1:**
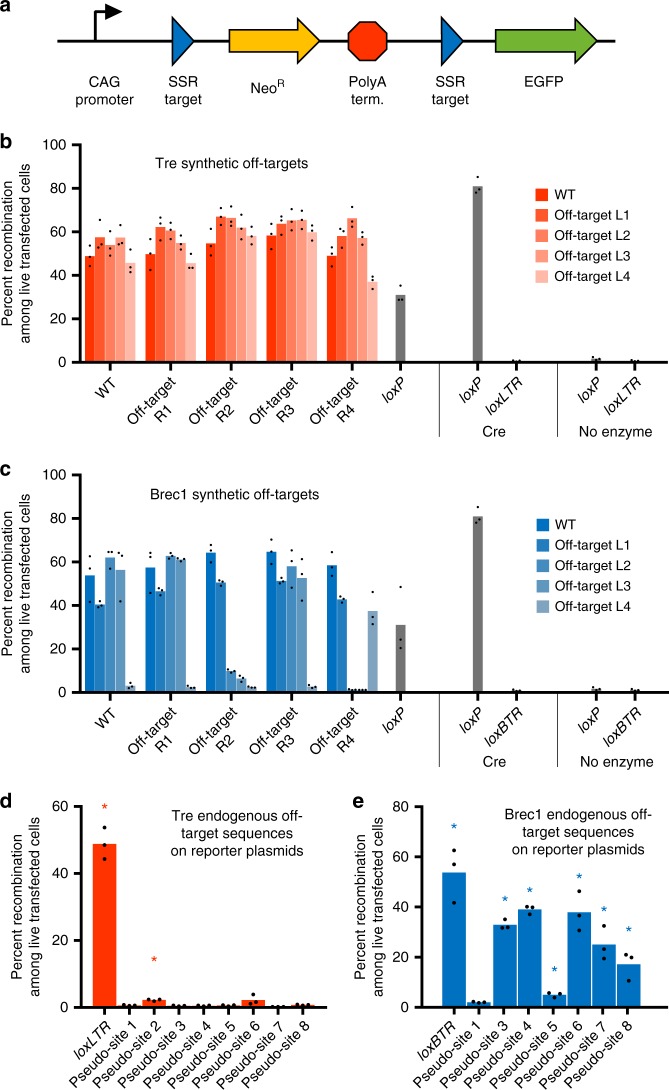
Fig. 1

**Table 1:**
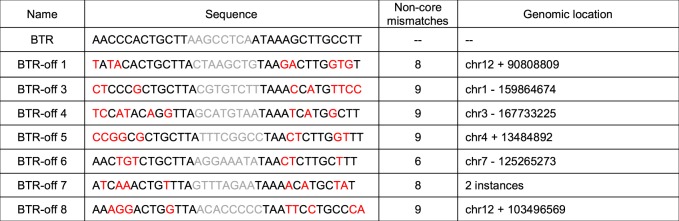
Human genomic off-target substrates for Brec1

Human genomic off-target Brec1. Mismatches relative to *loxBTR* (red) and core sequences (gray) are highlighted.

