# Supplementary material for: High-resolution specificity profiling and off-target prediction for site-specific DNA recombinases
Source: Nat Commun. 2019 Apr 26;10:1937. doi: 10.1038/s41467-019-09987-0 (PMC6486577; doi:10.1038/s41467-019-09987-0)
Supplement: Supplementary file 1 — Supplementary Information [file 41467_2019_9987_MOESM1_ESM.pdf]

High-resolution specificity profiling and off-target prediction for site-specific DNA recombinases. Bessen, J., et al.

SUPPLEMENTARY INFORMATION

Supplementary Figure 1. Impact of library randomization on Rec-seq profile.

Supplementary Figure 2. Cre:*loxP* core-nucleotide specificity.

Supplementary Figure 3. Rec-seq reaction parameter optimization.

Supplementary Figure 4. Rec-seq profile of purified versus commercial Cre.

Supplementary Figure 5. Quality score calculation.

Supplementary Figure 6. Asymmetric binding preference for Cre:*loxP.*

Supplementary Figure 7. Ala-substituted Cre variants.

Supplementary Figure 8. Impact of N-terminal mutations on Cre:*loxP* DNA specificity.

Supplementary Figure 9. Brec1 activity on previously-reported off-target sequences.

Supplementary Figure 10. Flow cytometry gating strategy.

Supplementary Table 1. κ_{avg} values for Rec-seq experiments.

Supplementary Table 2. Synthetic off-target substrates for Tre.

Supplementary Table 3. Synthetic off-target substrates for Brec1.

Supplementary Table 4. Human genomic off-target substrates for Tre.

Supplementary Table 5. Human genomic off-target substrates for Brec1.

Supplementary Table 6. Previously reported Brec1 off-target sequences.

Supplementary Table 7. Student’s t-test significance values for Rec-seq experiments.

Supplementary Table 8. Student’s paired t-test significance values for Rec-seq experiments.

Supplementary Table 9. Mann-Whitney U test significance values for Rec-seq experiments.

Supplementary Note 1. Rec-seq enrichment score derivation.

Supplementary Note 2. Rec-seq oligonucleotide sequences.

Supplementary Note 3. Amino acid sequences for Cre, Tre, Brec1, Dre, VCre, and BxB1.

Supplementary Note 4. Primers used in this study.

Supplementary Methods. Detailed Rec-seq protocol.

Supplementary References.

Source data for all Supplemental Figures are provided as a Source Data file.

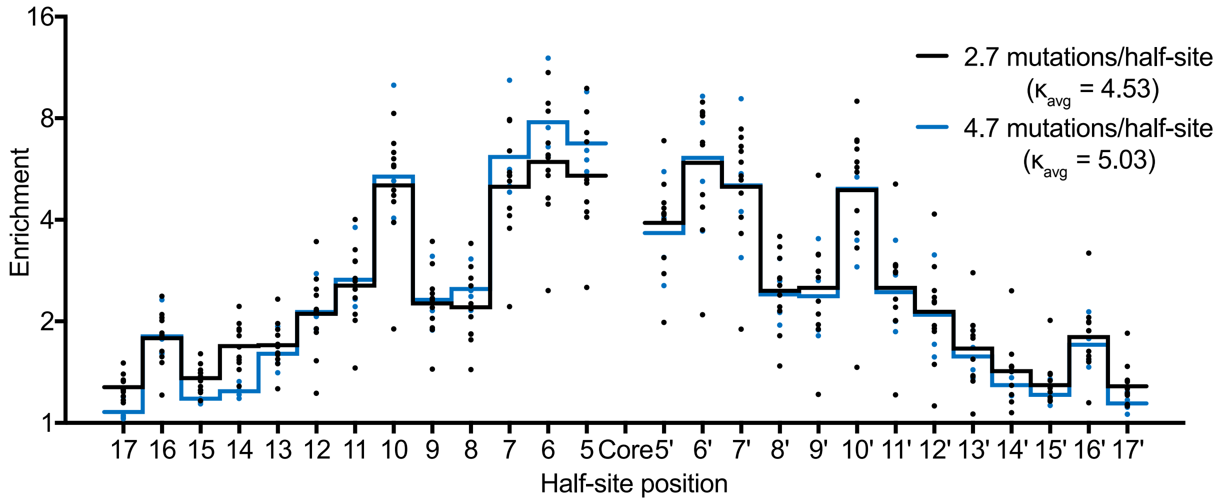

Supplementary Figure 1. Impact of library randomization on Rec-seq profile. Rec-seq profile for wild-type Cre on *loxP* using different levels of *loxP* library randomization. Values represent the geometric mean of n=11 (2.7 mutations/half-site) or n=4 (4.7 mutations/half-site) independent replicates (dots) conducted at 37 °C for 30 minutes at a 1:3 protein:DNA ratio. The differences between Cre enrichment on the two libraries were not significant ($p > 0.05$).

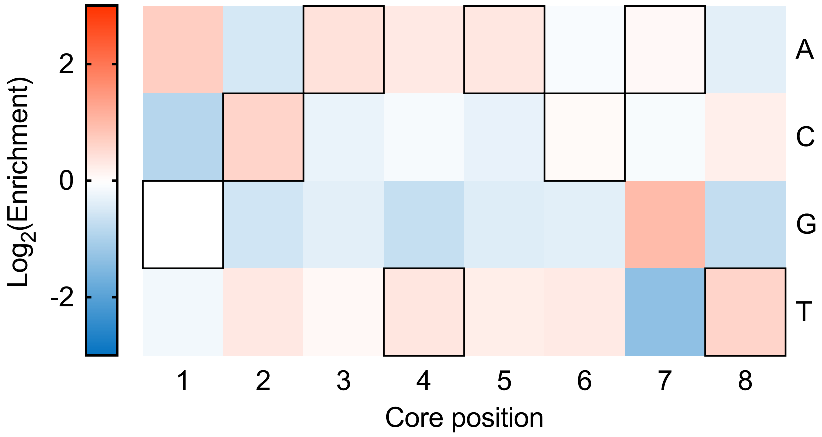

Supplementary Figure 2. Cre:*loxP* core-nucleotide specificity. Heat map of Rec-seq enrichment values for wild-type Cre showing the $\log_2$ of the enrichment value for each nucleotide at each position in the *loxP* core, relative to the canonical base for the forward orientation (black outline). Wild-type Cre was exposed to *loxP* library oligonucleotides in which the half-sites were held constant and the core nucleotides were unbiasedly randomized. Values represent the geometric mean of n=3 independent replicates conducted at 37 °C for 30 minutes at a 1:3 protein:DNA ratio.

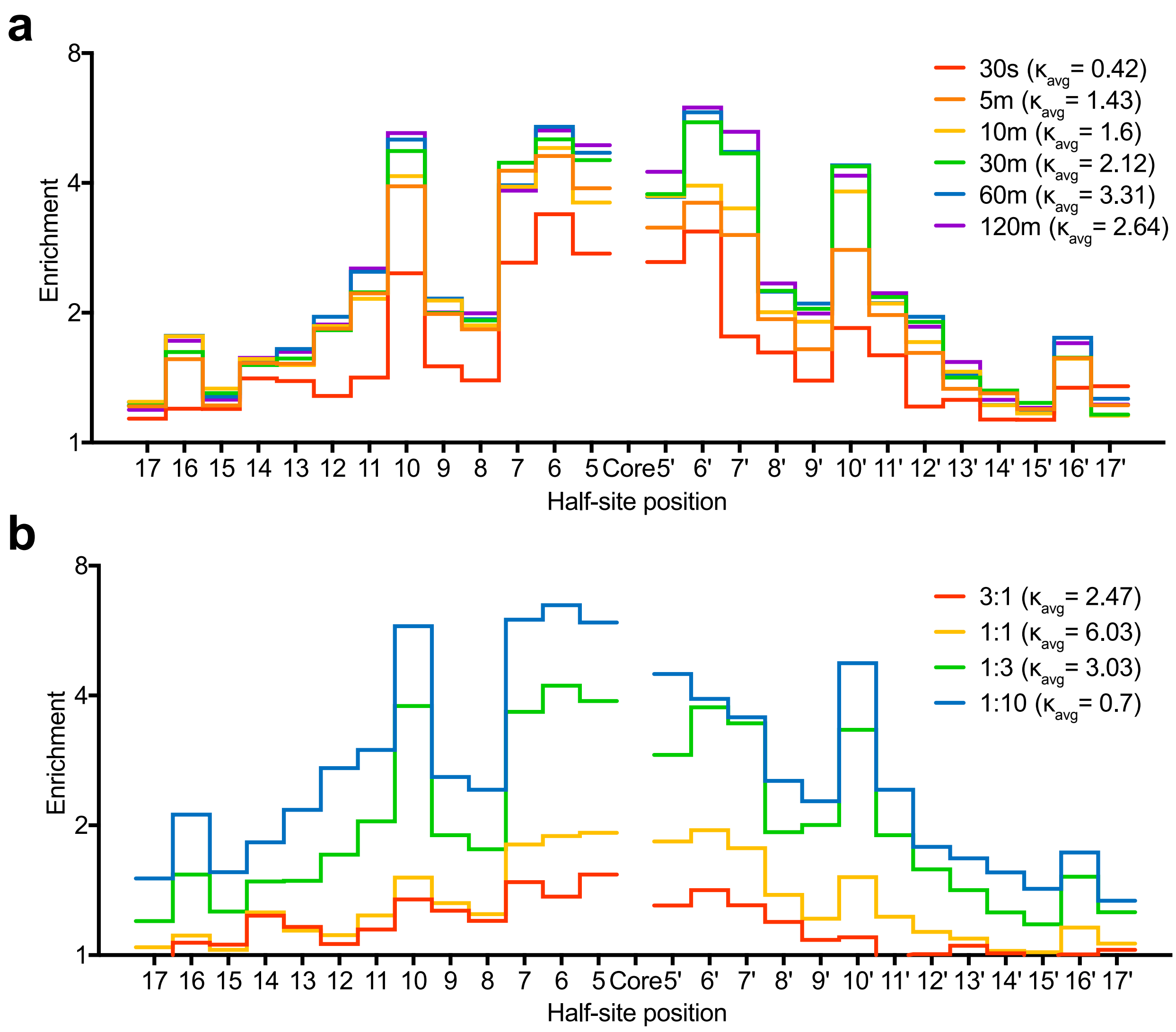

Supplementary Figure 3. Rec-seq reaction parameter optimization. Impact of reaction time (**a**) and protein:DNA ratio (**b**) on Rec-seq specificity profile for wild-type Cre reacted with *loxP* substrate. For part (**a**), all reactions were carried out at a 1:3 protein:DNA ratio. For part (**b**), all reactions were carried out for 30 minutes at 37 °C. Values represent the geometric mean of three independent replicates.

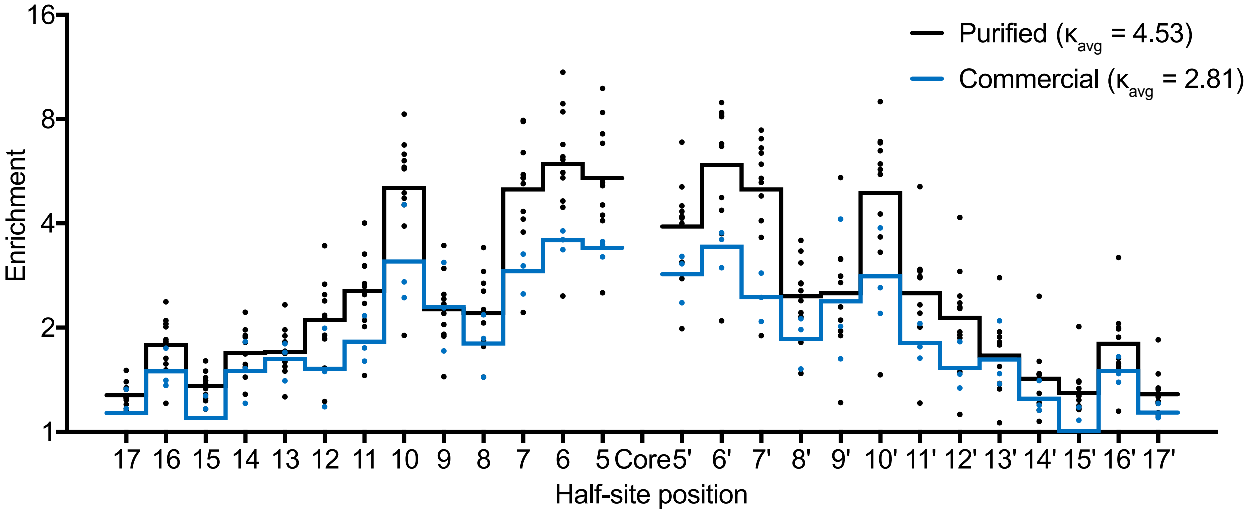

Supplementary Figure 4. Rec-seq profile of purified versus commercial Cre. Rec-seq profile for purified and commercially available wild-type Cre enzyme (New England Biolabs) on randomized *loxP* substrates. Values represent the geometric mean of n=11 (purified) or n=3 (commercial) independent replicates (dots) conducted at 37 °C for 30 minutes at a 1:3 protein:DNA ratio. The differences between commercial and purified Cre were not significant for any nucleotide position or along the full *loxP* site (p >> 0.05).

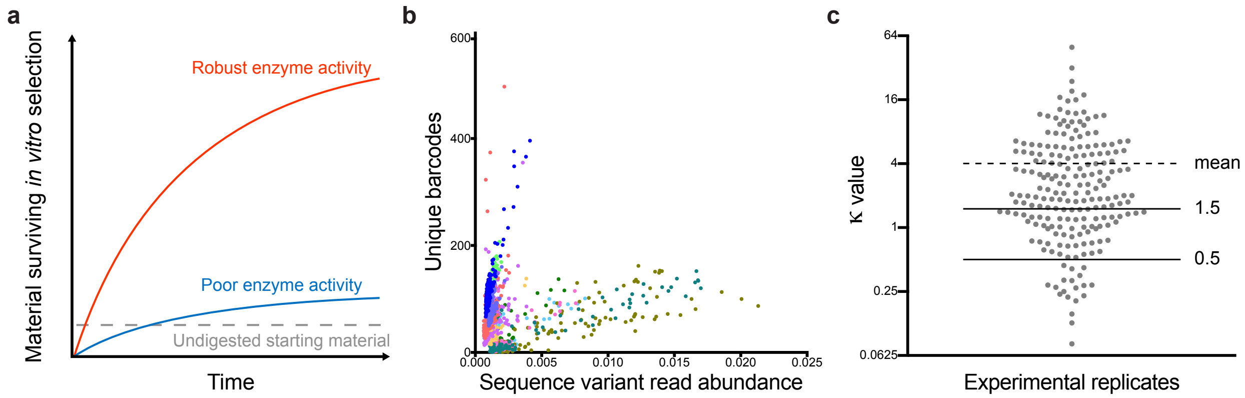

Supplementary Figure 5. Quality score calculation. a, Model for the effect of *in vitro* enzyme activity on apparent SSR specificity. For each experiment, a background level of undigested starting library is present (gray dashed line). This background undigested material is not distinguished from genuine recombination products that survive the *in vitro* selection. Robust enzyme activity produces an excess of genuine recombined products (red line), but poorly-active enzymes (blue line) or shortened reaction times produce lower levels of recombined products that can be similar to the level of background undigested starting material. **b**, To quantify the extent to which apparent specificity of an SSR is affected by its *in vitro* activity, we plotted the fractional abundance of each DNA sequence variant versus the number of unique barcodes for that variant. For DNA sequences with an absolute abundance of 800 or fewer (well below 4,096, the maximum number of unique barcodes), we assumed that each unique barcoded sample represented an independent recombination event. We expect that signal derived from few recombination events or amplification of undigested starting material would have relatively few unique barcodes for a given DNA sequence variant. We plotted the fractional abundance, as opposed to the absolute abundance, of each DNA sequence variant to correct for the effect of sequencing depth. The quality score κ is the slope of the best-fit line for the plot described above, divided by 10^4 for ease of comparison between experiments. The value κ_{avg} was calculated for each SSR variant by averaging the κ values for each experimental replicate. Exemplary data from 11 replicates of wild-type Cre reacted with *loxP* substrate at a 1:3 protein:DNA ratio for 30 minutes at 37 °C (colored dots) are shown. κ_{avg} values for each SSR variant can be found in Supplementary Table 1. **c,** Scatter plot showing the distribution of κ values for all Rec-seq experimental replicates on a $\log_2$ axis. We considered experiments to be well-powered if κ_{avg} values exceeded 1.5, moderately influenced by background signal for κ_{avg} values between 1.5 and 0.5, and heavily influenced by background signal for κ_{avg} values below 0.5.

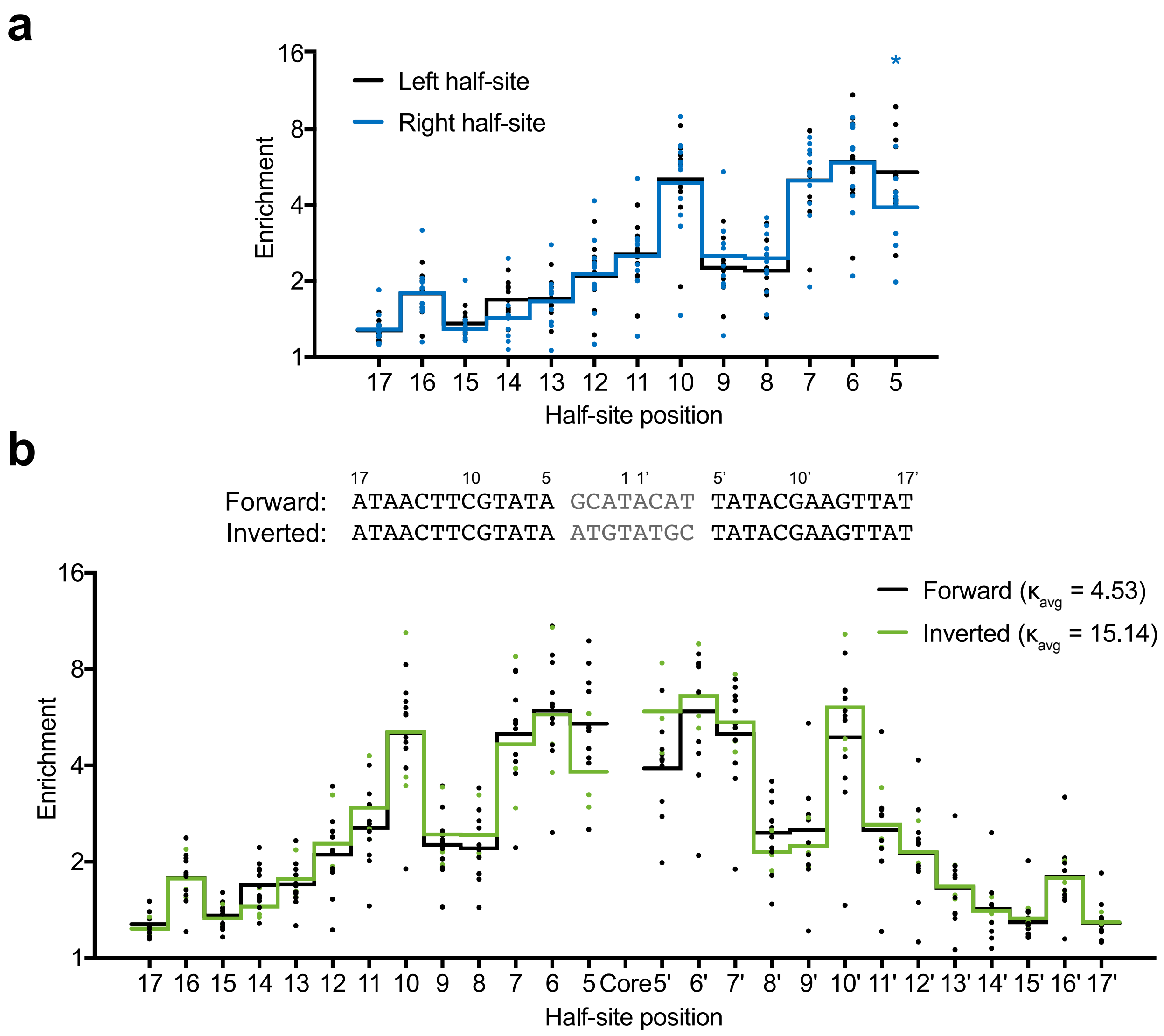

Supplementary Figure 6. Asymmetric binding preference for Cre:*loxP*. a, Superimposition of the left and right half-site enrichment profiles for purified wild-type Cre on *loxP* library oligonucleotides. Significant differences ($p \leq 0.05$; asterisks) between the log-enrichment values of the left and right half-sites were calculated using a paired t-test. **b**, Rec-seq of wild-type Cre on *loxP* library oligonucleotides with the core sequence in the forward or inverted direction. Values represent the geometric mean of n=11 or n=3 (inverted core) independent replicates (dots) conducted at 37 °C for 30 minutes at a 1:3 protein:DNA ratio.

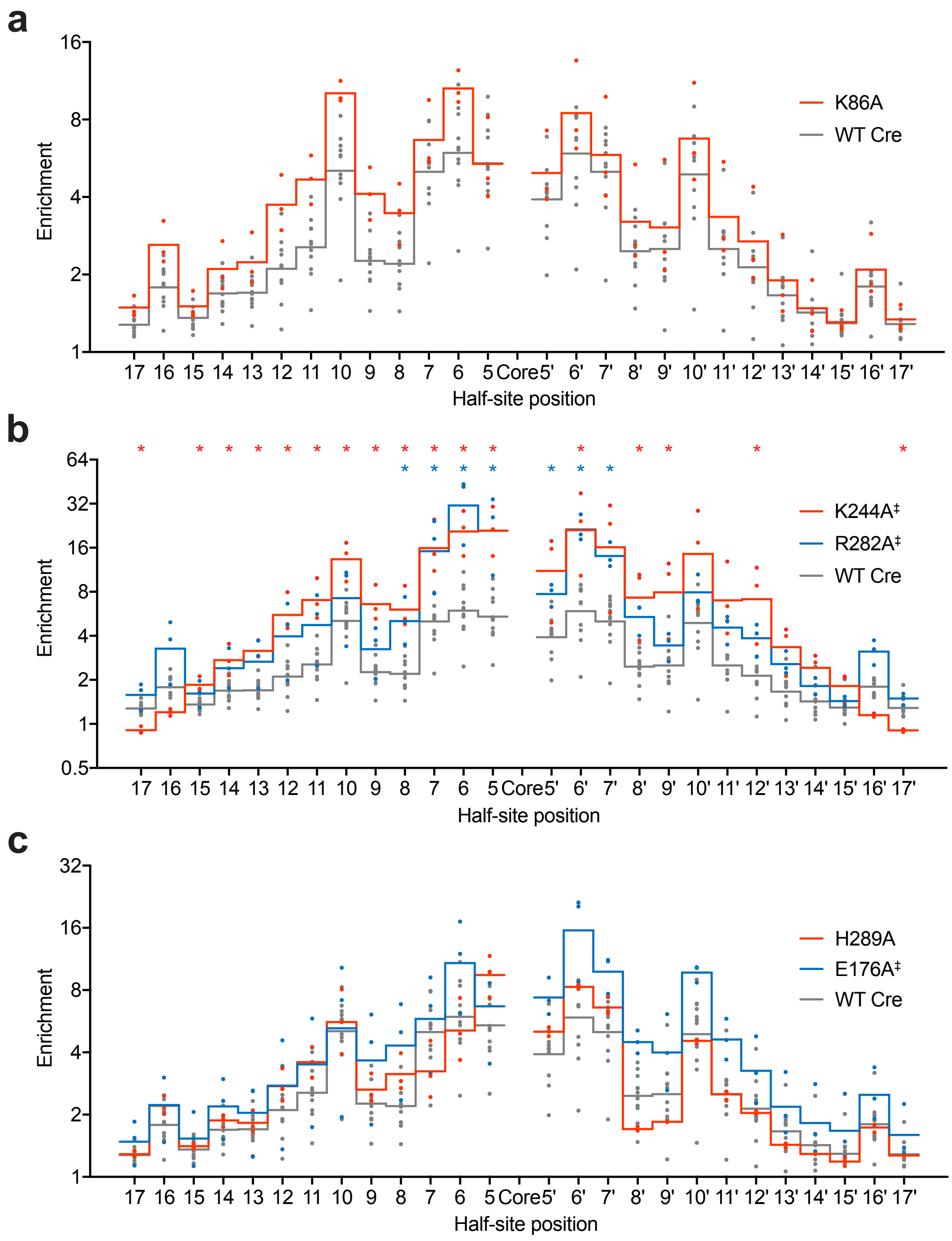

Supplementary Figure 7. Ala-substituted Cre variants. Rec-seq profiles for Ala-substituted variants of Cre (colored lines) that demonstrated (**a**) unchanged log-enrichment profiles or (**b**) globally increased specificity for *loxP* relative to wild-type Cre (gray line). **c,** Rec-seq profiles for Ala-substituted variants of Cre at residues that are highly conserved among tyrosine recombinases[1]. Values represent the geometric mean of n=11 (wild-type Cre) or n=3 independent replicates (dots) conducted at 37 °C for 30 minutes at a 1:3 protein:DNA ratio. Significant differences ($p \leq 0.05$) relative to wild-type Cre at individual nucleotides (colored asterisks) and across the full log-enrichment profile (‡) are indicated.

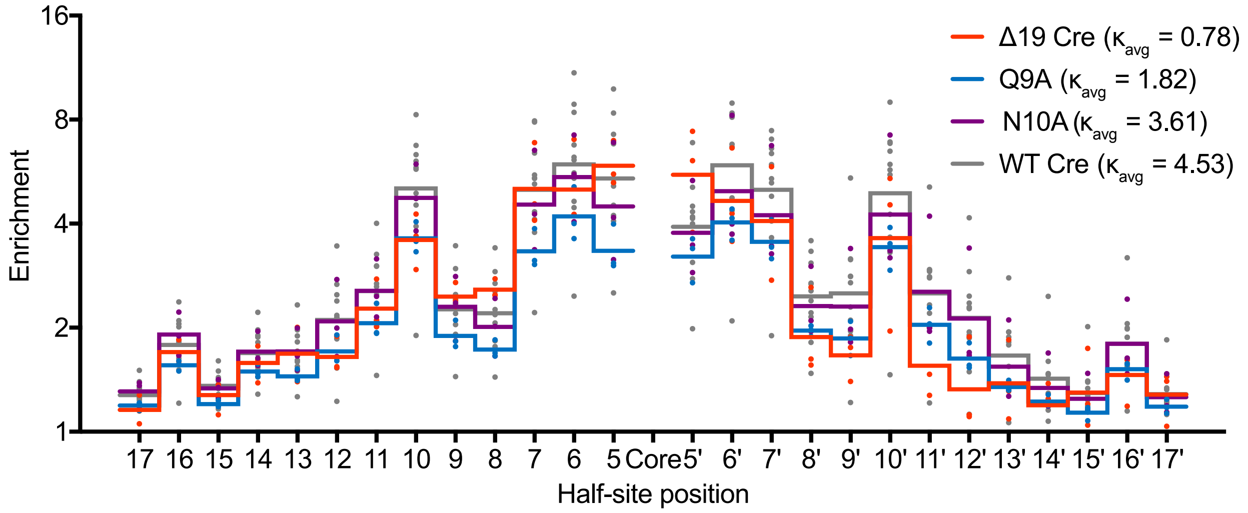

Supplementary Figure 8. Impact of N-terminal mutations on Cre:*loxP* DNA specificity. Rec-seq profiles for the N-terminal truncation (colored lines) relative to wild-type Cre (gray line). Values represent the geometric mean of n=11 (wild-type Cre) or n=3 independent replicates (dots) conducted at 37 °C for 30 minutes at a 1:3 protein:DNA ratio, except for N10A (1:1 molar ratio). The differences between N-terminal variants and wild-type Cre were not significant for any nucleotide position or along the full *loxP* site ($p >> 0.05$).

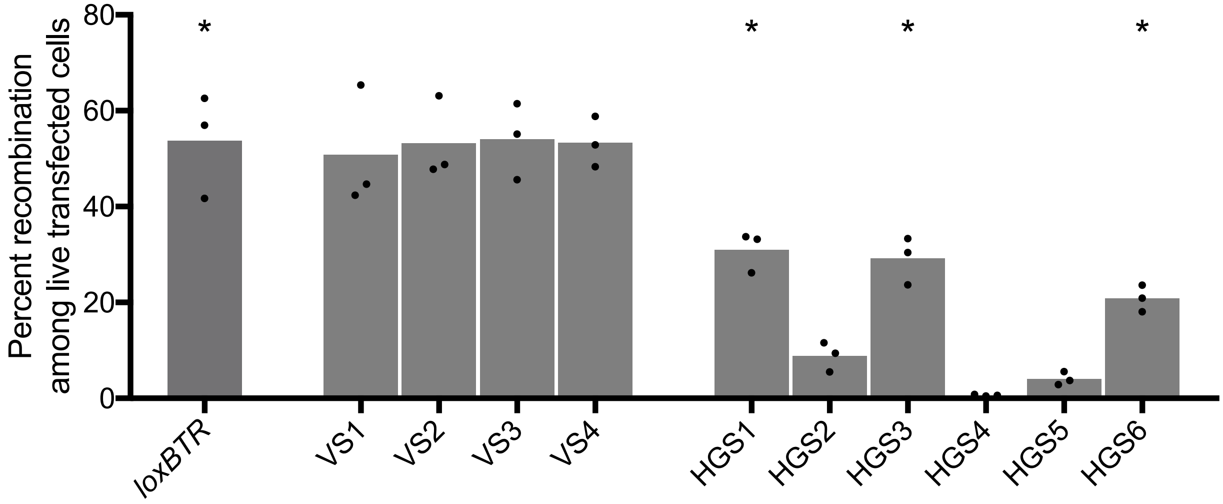

Supplementary Figure 9. Brec1 activity on previously-reported off-target sequences. Cells were transfected with Brec1 expression plasmid and a reporter plasmid bearing recombinase targets flanking a poly-A terminator that blocks *EGFP* transcription. Brec1 activity on *loxBTR*, singly-mismatched substrates (VS1-4), and potential genomic pseudo-sites (HGS1-6) was measured as the fraction of cells exhibiting EGFP fluorescence. The percentage of EGFP-positive cells shown is of transfected cells (determined by gating for the presence of co-transfected plasmid constitutively expressing mCherry) and 10,000 live events were recorded for each experiment. Data are represented as the mean (bars) of three independent biological replicates (dots). For HGS1-6, significant differences ($p \leq 0.05$) relative to no-enzyme control samples are indicated (asterisks). Statistical significance was not determined for VS1-4.

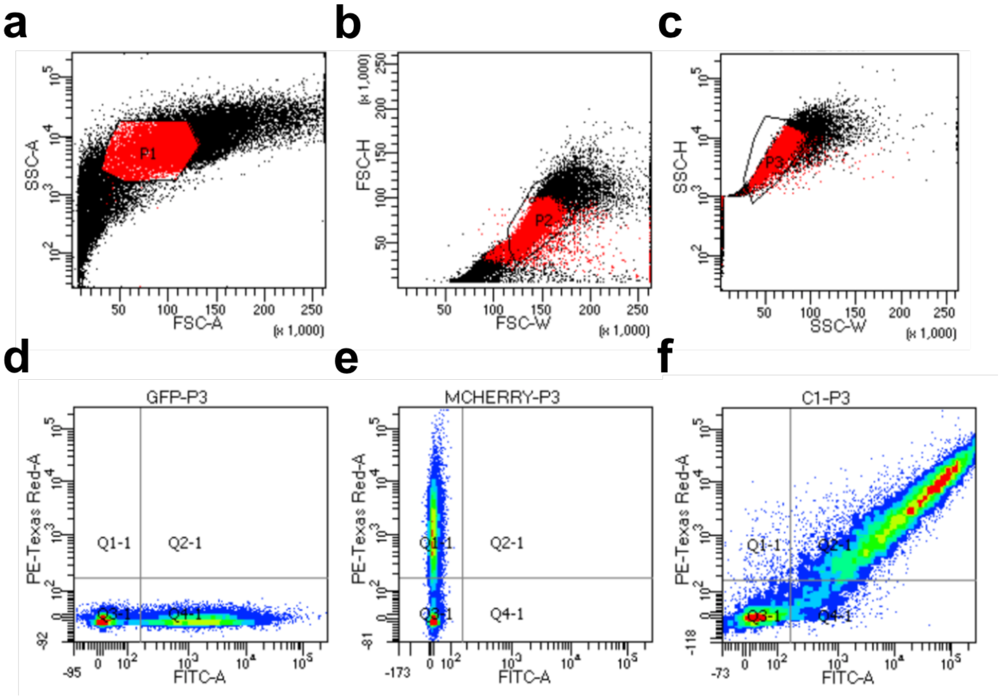

Supplementary Figure 10. Flow cytometry gating strategy. a-c, Gates for the live-cell population were constructed by size selection to remove debris and clumps of multiple cells. **d-f,** Densitometry plots depict FITC (GFP) and PE-Texas Red (mCherry) signal for control cells transfected with GFP-expressing plasmid only (**d**), mCherry-expressing plasmid only (**e**), or cells transfected with plasmids expressing the mCherry co-transfection marker, wild-type Cre, and a reporter plasmid bearing the *loxP* target. The percentage of EGFP-positive was calculated among live, transfected cells (determined by the presence of co-transfected mCherry plasmid) and 10,000 live events were recorded for each experiment.

Supplementary Table 1. κ_{avg} values for Rec-seq experiments.

Enzyme Variant	κ_{avg}
Brec1	0.28
Bxb1 attB	1.01
Bxb1 attP*	2.36
Δ19 Cre	0.78
Dre	1.49
E176A	1.41
E262A	1.34
H289A	1.09
K244A	3.48
K43A	1.61
K86A	2.32
M44A	4.90
N10A	3.61
Q90/94A	15.21
Q90A	7.40
Q94A	6.36
Q9A	1.82
R259A	5.61
R282A	11.20
Tre	5.17
VCre	1.82
WT Cre	4.53
WT Cre (4.7 mut./half-site)	5.03
WT Cre (inv. core)	15.14
WT Cre (commercial)	2.81

*κ_{avg} values for experiments with Bxb1 attP – L1 randomized oligonucleotides could not be calculated, as the unique molecular identifier was omitted due to DNA synthesis size limits.

Supplementary Table 2. Synthetic off-target substrates for Tre.

Name	Left half-site	Fold-enrichment
LTR	ACAACATCCTATTACAC	2.32
L1	ACAACATAATATTACAC	9.39
L2	ACAACTTGCTATTACAC	10.37
L3	CCAACATTCTATTACAC	10.32
L4	ACAACATTCTATAACAC	3.58

Name	Right half-site	Fold-enrichment
LTR	CCTATATGCCAACATGG	3.77
R1	CCTATATGCCAAGTTGG	17.57
R2	CCTATATACCAACTTGG	13.62
R3	CCTATATGGCAACTTGG	8.58
R4	CCTATATGCCAACAATA	> 39.0

Synthetic Tre substrates and fold-enrichment relative to input-library abundance. Mismatches relative to *loxLTR* (red) and core sequences (gray) are highlighted. Off-target R4 was not detected in sequencing of the pre-selection library, so the fold enrichment was calculated on the basis of the theoretical abundance of a triply-mutated sequence in the synthesized library.

Supplementary Table 3. Synthetic off-target substrates for Brec1.

Name	Left half-site	Fold-enrichment
BTR	AACCCACTGCTTAAGCC	3.10
L1	AACCCTCCGCTTAAGCC	14.74
L2	AACGCACTGTTTAAGCC	6.04
L3	AACCCACAGATTAAGCC	6.36
L4	AACCCCCTGATTAAGCC	7.19

Name	Right half-site	Fold-enrichment
BTR	TCAATAAAGCTTGCCTT	3.78
R1	TCAATAAACCTTGGCTT	6.03
R2	TCAATAATGCATGCCTT	17.01
R3	TCAATAAAGCTTGTATT	2.73
R4	TCAATAATGGGTGCCTT	> 159.8

Synthetic Brec1 substrates and fold-enrichment relative to input-library abundance. Mismatches relative to *loxBTR* (red) and core sequences (gray) are highlighted. Off-target R4 was not detected in sequencing of the pre-selection library, so the fold enrichment was calculated on the basis of the theoretical abundance of a triply-mutated sequence in the synthesized library.

Supplementary Table 4. Human genomic off-target substrates for Tre.

Name	Sequence	Non-core mismatches	Genomic location
LTR	ACAACATCCTATTACACCCTATATGCCAACATGG	--	--
LTR-off 1	TGAACTTAATATTTTTAATAGTATTGCAAATTGA	10	chr14 - 20878251, chr3 + 5904926
LTR-off 2	GCAACATGGTATTAGCTACTTTATCTCAATATGT	7	chr14 - 46653232, chr8 + 106953135
LTR-off 3	AAAACTTTATATTGAAGGAAATATGCCAAATGCA	9	chr3 + 53100634
LTR-off 4	TCAACCTTCTATTGATTTCTCTATTTCAATGGCT	10	chr7 + 43208243, chr4 + 135884591
LTR-off 5	AAAACATTATATTGAGTATAATATTCCAAAATAT	7	chr18 - 36924190, chr7 + 82176261
LTR-off 6	TGAACTTTATATTAATGGAATTATACCAAATGCA	11	11 instances
LTR-off 7	AGAACATGATATTACTCTCAATATCGCAAAAAGT	8	101 instances
LTR-off 8	GTAACATTATATTAATTTTAATATGACAAATCTA	10	6 instances

Human genomic off-targets for Tre. Mismatches relative to *loxLTR* (red) and core sequences (gray) are highlighted.

Supplementary Table 5. Human genomic off-target substrates for Brec1.

Name	Sequence	Non-core mismatches	Genomic location
BTR	AACCCACTGCTTAAGCCTCAATAAAGCTTGCCTT	--	--
BTR-off 1	TATACACTGCTTACTAAGCTGTAAGACTTGGTGT	8	chr12 + 90808809
BTR-off 2	ATGCCTCAGTTTATCCATCTGTAAAACATGGATT	11	23 instances
BTR-off 3	CTCCCGCTGCTTACGTGTCTTTAAACCATGTTCC	9	chr1 - 159864674
BTR-off 4	TCCATACAGGTTAGCATGTAATAAATCATGGCTT	9	chr3 - 167733225
BTR-off 5	CCGGCGCTGCTTATTTCGGCCTAACTCTTGGTTT	9	chr4 + 13484892
BTR-off 6	AACTGTCTGCTTAAGGAAATATAACTCTTGCTTT	6	chr7 - 125265273
BTR-off 7	ATCAAACTGTTTAGTTTAGAATAAAACATGCTAT	8	8 instances
BTR-off 8	AAAGGACTGGTTAACACCCCCTAATTCCTGCCCA	9	chr12 + 103496569

Human genomic off-targets for Brec1. Mismatches relative to *loxBTR* (red) and core sequences (gray) are highlighted.

Supplementary Table 6. Previously reported Brec1 off-target sequences.

Name	Sequence	Non-core mismatches	Genomic location
VS1	AACCCACTGCTTAAGCTTCAATAAAGCTTGCCTT	0	--
VS2	AACCCACCGCTTAAGCCTCAATAAAGCTTGCCTT	1	--
VS3	GACCCACTGCTTAAGCCTCAATAAAGCTTGCCTT	1	--
VS4	AGCCCACTGCTTAAGCCTCAATAAAGCTTGCCTT	1	--
HGS1	AAGCCCTTGCTTAAAAGGATTTAAAGAATGTTTA	8	4 instances
HGS2	AAATTATTGCTTATGAAGAAATAAAGCCAGCATT	7	chr4 – 138478069
HGS3	ATCCGATAGCTTATTTAATAATAAAGTTTGTATA	8	3 instances
HGS4	ATCCCACTGCTGAATATCCTCTAAAGCTTTCTGT	5	chr6 – 60734964, chr6 - 57983493
HGS5	GACGCATTCCTTATTCTTGAAAAAAGCTTGCATA	7	chr2 – 87894680, chrX + 144143849
HGS6	CACAATCTTCTTACACTGTAGTAAAGCTTGCTTG	7	4 instances

Off-target sequences previously assayed for Brec1 recognition[2]. Mismatches relative to *loxBTR* (red) and core sequences (gray) are highlighted.

Supplementary Table 7. Student's t-test significance values for Rec-seq experiments.

Enzyme variant	Half-site position	Bonferroni-corrected p value
Brec1	12	0.01256975
Brec1	10	0.00368103
Brec1	8	0.00178504
Brec1	5'	5.32E-05
Brec1	8'	0.00040415
K244A	17	0.00041764
K244A	15	0.01015747
K244A	14	0.03528123
K244A	13	0.00159779
K244A	12	0.00517888
K244A	11	0.00332736
K244A	10	0.04740163
K244A	9	0.0004642
K244A	8	0.00203837
K244A	7	0.0116214
K244A	6	0.01010555
K244A	5	0.00385061
K244A	6'	0.04993283
K244A	8'	0.00872169
K244A	9'	0.04073499
K244A	12'	0.01620585
K244A	17'	0.04073309
M44A	5	0.00532987
R259A	17	0.0101429
R259A	16	0.00133818
R259A	15	0.03089689
R259A	14	0.00731429
R259A	13	0.0157074
R259A	10	0.00075264
R259A	8	0.02012748
R259A	7	0.00397227
R259A	6	0.00257851
R259A	6'	0.0145799
R259A	10'	0.00639154
R259A	14'	0.04347332
R259A	16'	0.00305847
R282A	8	0.01524759
R282A	7	0.03535704
R282A	6	0.00173287
R282A	5	0.01078905
R282A	6'	0.01441939
R282A	7'	0.02595199
R282A	8'	0.01561694
Tre	17	0.00026323
Tre	15	0.00439622
Tre	12	0.03084838
Tre	10	2.46E-05
Tre	9	7.83E-05
Tre	5'	0.00270499
Tre	6'	0.00512937
Tre	14'	0.00092103

Significance of log-enrichment values was calculated by performing the Student's t-test assuming equal variance for each individual position of each SSR variant relative to wild-type Cre, and the effect of multiple comparisons was counteracted using the Bonferroni correction.

Supplementary Table 8. Paired student's t-test significance values for Rec-seq experiments.

Enzyme variant	Half-site position	Bonferroni-corrected p-value
WT Cre	5/5'	0.02522168

Significance of log-enrichment values between the left and right half-sites of wild-type Cre was calculated by performing a paired t-test, and the effect of multiple comparisons was counteracted using the Bonferroni correction.

Supplementary Table 9. Mann-Whitney U test significance values for Rec-seq experiments.

Enzyme variant	Bonferroni-corrected p-value
Brec1	0.01583792
E176A	1.03E-11
K244A	5.39E-17
Q90/94A	1.31E-05
Q90A	0.00019859
Q94A	0.01969353
R259A	7.17E-10
R282A	2.99E-11
Tre	3.49E-6

Significance of full substrate log-enrichment profiles was calculated using the two-sided Mann-Whitney U test. We compared the absolute value of the residuals for wild-type Cre and each enzyme variant, and applied the Bonferroni correction.

Supplementary Note 1. Rec-seq enrichment score derivation.

The enrichment score for Rec-seq experiments was derived as follows. Let p_i be the recombination probability of substrates containing the canonical nucleotide at position i and let $q_i = 1 - p_i$ be the recombination probability of recombination for substrates not containing the non-cognate nucleotide at position i, assuming an unbiased input library. The enrichment score, measuring the preference of the enzyme for the canonical nucleotide at position i, is expressed by the odds ratio $r_i = p_i / q_i$.

Let α_i denotes a frequency of the canonical nucleotide at a position i of the substrate and let $\beta_i = 1 - \alpha_i$ be the frequency of the other three nucleotides at that position in the input library. The number of reads that contain (A_i) or do not contain (B_i) the canonical nucleotide at position i after recombinase treatment of the biased library conforms to the binomial distribution

$$P(A,B;p,q) = \binom{A+B}{A}\left(\frac{\alpha p}{\alpha p + \beta q}\right)^{A}\left(\frac{\beta q}{\alpha p + \beta q}\right)^{B}$$

or, when written in terms of ratio the r_i,

$$P(A,B;r) = \binom{A+B}{A}\left(\frac{\alpha r}{\alpha r + \beta}\right)^{A}\left(\frac{\beta}{\alpha r + \beta}\right)^{B}.$$

For simplicity, we have dropped the index i from all equations. For a given input library composition (α_i, β_i) and the composition observed after recombination (A_i, B_i), the maximum-likelihood estimate of the enrichment score can be obtained, using a method described previously[3], when the parameter of the binomial distribution is equal to the fraction of the observed reads, *e.g*:

$$\frac{\alpha r}{\alpha r + \beta} = \frac{A}{A+B}$$

which, when simplified, yields the formula for the enrichment score:

$$r = \frac{\frac{A}{B}}{\frac{\alpha}{\beta}}.$$

Supplementary Note 2. Rec-seq oligonucleotide sequences.

All oligonucleotides were ordered from Integrated DNA Technologies. 'N' signifies a mixture of A, T, G, or C, synthesized using the standard machine-mixed pool of phosphoramidites. Nucleotides in bold were synthesized using a customized mixture containing 79% bolded base and 7% each of the other three bases, for an average 2.7 mutations per 13-bp half-site. (Libraries containing 64% of the wild-type base and 12% each of the other three bases were also synthesized for *loxP*, for an average 4.7 mutations per 13-bp half-site.)

In the table, the hairpin sequence (red), post-recombination primer-binding sequences (blue), unique molecular identifier (UMI) barcode (green), and pre-recombination library-amplification binding sequences (purple) are highlighted.

The naming convention for library oligonucleotides is as follows:

- '1' represents the left-sided hairpin, while '2' represents the right-sided hairpin.
- 'left' signifies that the left half-site of the substrate is randomized, whereas 'right' signifies the right half-site is randomized.

Name	Sequence
F.Trans.Miseq	ACACTCTTTCCCTACACGACGCTCTTCCGATCTNNNNACGTAGTACTCGGTCTC
R.Trans.Miseq	TGGAGTTCAGACGTGTGCTCTTCCGATCTGTCCGGCCGTATTGTGTCC
F.presel.Miseq	ACTCTTTCCCTACACGACGCTCTTCCGATCTNNNNTGTCTTACCATGGGC
R.presel.Miseq	GGAGTTCAGACGTGTGCTCTTCCGATCTTCACGTACTCTCGAGCG
Trans.loxP.1	TCACGTACTCTCGAGCGNNATAACTTCGTATAATGTATGCTATACGAAGTTATNNGCNNNNNNG<br>AGACCGAGTACTACGTAATTACGTAG
Trans.loxP.2	TGTCTTACCATGGGCNNATAACTTCGTATAGCATACATTATACGAAGTTATNNCGATATAGGGAC<br>ACAATACGGCCGGACAATTGTCCGG
Trans.loxPleft.1	TCACGTACTCTCGAGCGNNATAACTTCGTATAATGTATGC**TATACGAAGTTAT**NNGCNNNNNNG<br>AGACCGAGTACTACGTAATTACGTAG
Trans.loxPright.2	TGTCTTACCATGGGCNNATAACTTCGTATAGCATACAT**TATACGAAGTTAT**NNCGATATAGGGAC<br>ACAATACGGCCGGACAATTGTCCGG
Trans.loxP.invcore.1	TCACGTACTCTCGAGCGNNATAACTTCGTATAGCATACATTATACGAAGTTATNNGCNNNNNNG<br>AGACCGAGTACTACGTAATTACGTAG
Trans.loxP.invcore.2	TGTCTTACCATGGGCNNATAACTTCGTATAATGTATGCTATACGAAGTTATNNCGATATAGGGAC<br>ACAATACGGCCGGACAATTGTCCGG
Trans.loxPleft.invcore.1	TCACGTACTCTCGAGCGNNATAACTTCGTATAGCATACAT**TATACGAAGTTAT**NNGCNNNNNNG<br>AGACCGAGTACTACGTAATTACGTAG
Trans.loxPright.invcore.2	TGTCTTACCATGGGCNNATAACTTCGTATAATGTATGC**TATACGAAGTTAT**NNCGATATAGGGAC<br>ACAATACGGCCGGACAATTGTCCGG
Trans.loxP.CORE.1	TCACGTACTCTCGAGCGNNATAACTTCGTATANNNNNNNNTATACGAAGTTATNNGCNNNNNNG<br>AGACCGAGTACTACGTAATTACGTAG
Trans.loxP.CORE.2	TGTCTTACCATGGGCNNATAACTTCGTATANNNNNNNNTATACGAAGTTATNNCGATATAGGGA<br>CACAATACGGCCGGACAATTGTCCGG
Trans.loxLTR.1	TCACGTACTCTCGAGCGNNCCATGTTGGCATATAGGGTGTAATAGGATGTTGTNNGCNNNNNN<br>GAGACCGAGTACTACGTAATTACGTAG
Trans.loxLTR.2	TGTCTTACCATGGGCNNACAACATCCTATTACACCCTATATGCCAACATGGNNCGATATAGGGA<br>CACAATACGGCCGGACAATTGTCCGG
Trans.loxLTRleft.1	TCACGTACTCTCGAGCGNNCCATGTTGGCATATAGGGTGT**AATAGGATGTTGT**NNGCNNNNNN<br>GAGACCGAGTACTACGTAATTACGTAG
Trans.loxLTRright.2	TGTCTTACCATGGGCNNACAACATCCTATTACACCCTA**TATGCCAACATGG**NNCGATATAGGGA<br>CACAATACGGCCGGACAATTGTCCGG
Trans.loxBTR.1	TCACGTACTCTCGAGCGNNAAGGCAAGCTTTATTGAGGCTTAAGCAGTGGGTTNNGCNNNNNN<br>GAGACCGAGTACTACGTAATTACGTAG
Trans.loxBTR.2	TGTCTTACCATGGGCNNAACCCACTGCTTAAGCCTCAATAAAGCTTGCCTTNNCGATATAGGGA<br>CACAATACGGCCGGACAATTGTCCGG
Trans.loxBTRleft.1	TCACGTACTCTCGAGCGNNAAGGCAAGCTTTATTGAGGCT**TAAGCAGTGGGTT**NNGCNNNNNN<br>GAGACCGAGTACTACGTAATTACGTAG

Trans.loxBTRright.2	TGTCTTACCATGGGCNNAACCCACTGCTTAAGCCTCAA**TAAAGCTTGCCTT**NNCGATATAGGGACACAATACGGCCGGACAATTGTCCGG
Trans.rox.1	TCACGTACTCTCGAGCGNNTAACTTTAAATAATTGGCATTATTTAAAGTTANNGCNNNNNNGAGACCGAGTACTACGTAATTACGTAG
Trans.rox.2	TGTCTTACCATGGGCNNTAACTTTAAATAATGCCAATTATTTAAAGTTANNCGATATAGGGACACAATACGGCCGGACAATTGTCCGG
Trans.roxleft.1	TCACGTACTCTCGAGCGNNTAACTTTAAATAATTGGCAT**TATTTAAAGTTA**NNGCNNNNNNGAGACCGAGTACTACGTAATTACGTAG
Trans.roxright.2	TGTCTTACCATGGGCNNTAACTTTAAATAATGCCAAT**TATTTAAAGTTA**NNCGATATAGGGACACAATACGGCCGGACAATTGTCCGG
Trans.loxV.1	TCACGTACTCTCGAGCGNNTCAATTTCCGAGAATGACAGTTCTCAGAAATTGANNGCNNNNNNGAGACCGAGTACTACGTAATTACGTAG
Trans.loxV.2	TGTCTTACCATGGGCNNTCAATTTCTGAGAACTGTCATTCTCGGAAATTGANNCGATATAGGGACACAATACGGCCGGACAATTGTCCGG
Trans.loxVleft.1	TCACGTACTCTCGAGCGNNTCAATTTCCGAGAATGACAGT**TCTCAGAAATTGA**NNGCNNNNNNGAGACCGAGTACTACGTAATTACGTAG
Trans.loxVright.2	TGTCTTACCATGGGCNNTCAATTTCTGAGAACTGTCAT**TCTCGGAAATTGA**NNCGATATAGGGACACAATACGGCCGGACAATTGTCCGG
Trans.BxB1attP.1	TCACGTACTCTCGAGCGNNGTCGGGGTTTGTACCGTACACCACTGAGACCGCGGTGGTTGACCAGACAAACCACGACNNGCNNNNNNGAGACCGAGTACTACGTAATTACGTAG
Trans.BxB1attP.2	TGTCTTACCATGGGCNNGTCGTGGTTTGTCTGGTCAACCACCGCGGTCTCAGTGGTGTACGGTACAAACCCCGACNNCGGGACACAATACGGCCGGACAATTGTCCGG
Trans.BxB1attP.left.1	TCACGTACTCTCGAGCGNNGGTTTGTACCGTACACCACTGAGAC**CGCGGTGGTTGACCAGACAAACC**NNGCGAGACCGAGTACTACGTAATTACGTAG
Trans.BxB1attP.right.2	TGTCTTACCATGGGCNNGGTTTGTCTGGTCAACCACCGCGGT**CTCAGTGGTGTACGGTACAAACC**NNCGGGACACAATACGGCCGGACAATTGTCCGG
Trans.BxB1attB.1	TCACGTACTCTCGAGCGNNGCCCGGATGATCCTGACGACGGAGACCGCCGTCGTCGACAAGCCGGCCGANNGCNNNNNNGAGACCGAGTACTACGTAATTACGTAG
Trans.BxB1attB.2	TGTCTTACCATGGGCNNTCGGCCGGCTTGTCGACGACGGCGGTCTCCGTCGTCAGGATCATCCGGGCNNCGGGACACAATACGGCCGGACAATTGTCCGG
Trans.BxB1attB.left.1	TCACGTACTCTCGAGCGNNATGATCCTGACGACGGAGAC**CGCCGTCGTCGACAAGCC**NNGCNNNNNNGAGACCGAGTACTACGTAATTACGTAG
Trans.BxB1attB.right.2	TGTCTTACCATGGGCNNGGCTTGTCGACGACGGCGGT**CTCCGTCGTCAGGATCAT**NNCGATATAGGGACACAATACGGCCGGACAATTGTCCGG

Supplementary Note 3. Amino acid sequences for Cre, Tre, Brec1, Dre, VCre, and Bxb1.

Within recombinase sequences, affinity tags (yellow) are highlighted. For mammalian-cell experiments involving Cre, Tre, and Brec1, the affinity tags were omitted.

Cre
MAHHHHHHGGSSNLLTVHQNLPALPVDATSDEVRKNLMDMFRDRQAFSEHTWKMLLSVCRSWAAWCK
LNNRKWFPAEPEDVRDYLLYLQARGLAVKTIQQHLGQLNMLHRRSGLPRPSDSNAVSLVMRRIRKENVD
AGERAKQALAFERTDFDQVRSLMENSDRCQDIRNLAFLGIAYNTLLRIAEIARIRVKDISRTDGGRMLIHIG
RTKTLVSTAGVEKALSLGVTKLVERWISVSGVADDPNNYLFCRVRKNGVAAPSATSQLSTRALEGIFEAT
HRLIYGAKDDSGQRYLAWSGHSARVGAARDMARAGVSIPEIMQAGGWTNVNIVMNYIRNLDSETGAMV
RLLEDGD

Tre
MAHHHHHHGGSSNLLTLHHSLPALPADATSDEVRKNLMDVFRDRPAFSEHTWEMLLSVCRSWAAWCKL
NNRKWFPAEPEDVRDYLLHLQARGLAVKTIQQHLCRLNMLHRRSGLPRPSDSNAVSLVMRRIRKENVDA
GERTKQALAFERTDFDQVRSLMENSDRCQDIRNLAFLGVAYNTLLRIAEIARIRVKDISRTDGGRMLIHIGR
TKTLVSTAGVEKALSLGVTKLVERWISVSGVADDPNNYLFCRVRRYGVAAPSATSQLSTYALQRIFEATH
RLIYGAKDDSGQRYLAWSGHSARVGAARDMARAGVSIPEIMQAGGWTTVNSVMNYIRNLDSETGAMVR
LLEDGD

Brec1
MHHHHHHENLYFQGAASMSILLTLHQSLSALLVDATSDEARKNLMDVLRDRQAFSERTWKVLLSVCRTW
AAWCKLNNRKWFPAEPEDVRDYLLHLQARGLAVNTILQHLAQLNMLHRRFGLPRPGDSDAVSLVMRRIR
RENVDAGERTKQALAFERTDFDQVRALMENSERGQDIRTLAL**P**GVAYNTLLRVSEIARIRIKDISRTDGGR
MLIHISRTKTLVSTAGVEKALSLGVTKLVERWISVSGVASDPNNYLFCQVRINGVAVPSATSRLSTDVLRKI
FEAAHRLIYGAKDGSGQRYLAWSGHSARVGAARDMARAGVSIAEIMQAGGWTTVESVMNYIRNLDSET
GAMVRLLEDGD

*Purified Brec1 contained a previously-unpublished Leu163Phe stabilizing mutation (bold), while the mammalian-cell experiments were performed with Brec1 as published.

Dre
MAHHHHHHGGSMSELIISGSSGGFLRNIGKEYQEAAENFMRFMNDQGAYAPNTLRDLRLVFHSWARWC
HARQLAWFPISPEMAREYFLQLHDADLASTTIDKHYAMLNMLLSHCGLPPLSDDKSVSLAMRRIRREAAT
EKGERTGQAIPLRWDDLKLLDVLLSRSERLVDLRNRAFLFVAYNTLMRMSEISRIRVGDLDQTGDTVTLHI
SHTKTITTAAGLDKVLSRRTTAVLNDWLDVSGLREHPDAVLFPPIHRSNKARITTTPLTAPAMEKIFSDAWV
LLNKRDATPNKGRYRTWTGHSARVGAAIDMAEKQVSMVEIMQEGTWKKPETLMRYLRRGGVSVGANS
RLMDS

VCre
MAHHHHHHGGSIENQLSLLGDFSGVRPDDVKTAIQAAQKKGINVAENEQFKAAFEHLLNEFKKREERYSP
NTLRRLESAWTCFVDWCLANHRHSLPATPDTVEAFFIERAEELHRNTLSVYRWAISRVHRVAGCPDPCL
DIYVEDRLKAIARKKVREGEAVKQASPFNEQHLLKLTSLWYRSDKLLLRRNLALLAVAYESMLRASELANI
RVSDMELAGDGTAILTIPITKTNHSGEPDTCILSQDVVSLLMDYTEAGKLDMSSDGFLFVGVSKHNTCIKP
KKDKQTGEVLHKPITTKTVEGVFYSAWETLDLGRQGVKPFTAHSARVGAAQDLLKKGYNTLQIQQSGRW
SSGAMVARYGRAILARDGAMAHSRVKTRSAPMQWGKDEKD

Bxb1
MRALVVIRLSRVTDATTSPERQLESCQQLCAQRGWDVVGVAEDLDVSGAVDPFDRKRRPNLARWLAFE
EQPFDVIVAYRVDRLTRSIRHLQQLVHWAEDHKKLVVSATEAHFDTTTPFAAVVIALMGTVAQMELEAIKE
RNRSAAHFNIRAGKYRGSLPPWGYLPTRVDGEWRLVPDPVQRERILEVYHRVVDNHEPLHLVAHDLNRR
GVLSPKDYFAQLQGREPQGREWSATALKRSMISEAMLGYATLNGKTVRDDDGAPLVRAEPILTREQLEA
LRAELVKTSRAKPAVSTPSLLLRVLFCAVCGEPAYKFAGGGRKHPRYRCRSMGFPKHCGNGTVAMAEW

DAFCEEQVLDLLGDAERLEKVWVAGSDSAVELAEVNAELVDLTSLIGSPAYRAGSPQREALDARIAALAA
RQEELEGLEARPSGWEWRETGQRFGDWWREQDTAAKNTWLRSMNVRLTFDVRGGLTRTIDFGDLQEY
EQHLRLGSVVERLHTGMSGGSHHHHHH

Supplementary Note 4. Primers used in this study.

All oligonucleotides were purchased from Integrated DNA Technologies.

Primers used for constructing pET vectors by LCR assembly[4]

Purpose	Name	Sequence
F pET sequencing primer	JB788	GAAACAAGCGCTCATGAGCCCGAA
R pET sequencing primer	JB789	GTCCCATTCGCCAATCCGGATATAG
F vector backbone (N-term His-tag)	JB1509	CTCGAGTCTGGTAAAGAAACCGCTG
R vector backbone (N-term His-tag)	JB1506	GCTCCCGCCGTGATGGTGGTGGTGA
F Cre insert	JB786	GGCGGGAGCAGCAATTTACTGACCGTACACCAAAATTTG
R Cre insert	JB204	TTAGTCGCCATCTTCCAGCAGG
Cre bridging oligo 1	JB787	TGGCGCATCACCACCACCATCACGGCGGGAGCAGCAATTTACTGACCG
Cre bridging oligo 2	JB1511	CGCCTGCTGGAAGATGGCGACTAACTCGAGTCTGGTAAAGAAACCGCTGC
F Tre insert	JB790	AGCAATTTACTGACCCTGCACCA
R Tre insert	JB204	see above
Tre bridging oligo 1	JB1512	GCATCACCACCACCATCACGGCGGGAGCAGCAATTTACTGACCCTGCACCA
Tre bridging oligo 2	JB1511	see above
F Brec1 insert	JB1508	AGCATCTTACTGACCCTTCACC
R Brec1 insert	JB204	see above
Brec1 bridging oligo 1	JB1510	TCACCACCACCATCACGGCGGGAGCAGCATCTTACTGACCCTTCACCAAAGCTTG
Brec1 bridging oligo 2	JB1511	see above
F Dre insert	JB1515	ATGTCTGAGCTGATTATTAGTGGTTCATC
R Dre insert	JB1516	TCAGCTATCCATCAGTCGAGAATTGG
Dre bridging oligo 1	JB1517	GCATCACCACCACCATCACGGCGGGAGCATGTCTGAGCTGATTATTAGTGGTTCATCTGG
Dre bridging oligo 2	JB1518	GGGAGCCAATTCTCGACTGATGGATAGCTGACTCGAGTCTGGTAAAGAAACCGCTGC
F VCre insert	JB1870	ATAGAGAATCAGCTAAGCTTACTGGG
R VCre insert	JB143	TTAGTCCTTTTCATCTTTGCCCCATTG
VCre bridging oligo 1	JB1871	GCATCACCACCACCATCACGGCGGGAGCATAGAGAATCAGCTAAGCTTACTGGG
VCre bridging oligo 2	JB1872	CAATGGGGCAAAGATGAAAAGGACTAACTCGAGTCTGGTAAAGAAACCGCTGC
F vector backbone (C-term His-tag)	JB1520	GGCGGATCCCATCACCACCACCAT
R vector backbone (C-term His-tag)	JB1519	ATGGTATATCTCCTTCTTAAAGTTAAACAAAATTATTTC
F BxB1 insert	JB82	GAGAGCCCTGGTAGTCATCC
R BxB1 insert	JB380	CGACATCCCGGTGTGTAGCCGTTCG
BxB1 bridging oligo 1	JB1523	GAAATAATTTTGTTTAACTTTAAGAAGGAGATATACCATGAGAGCCCTGGTAGTCATCCGCC
BxB1 bridging oligo 2	JB1524	CGAACGGCTACACACCGGGATGTCGGGCGGATCCCATCACCACCACCAT

Primers used for constructing Ala-substitutions by blunt end ligations

Purpose	Name	Sequence
F Q9A variant	JB1867	TTGCCTGCATTGCCGGT
R Q9A variant	JB1868	ATTGGCGTGTACGGTCAGTAAATTGCT
F N10A variant	JB1867	see above
R N10A variant	JB1869	GGCTTGGTGTACGGTCAGTAAATTGCT
F delta 19 variant	JB393	ACGAGTGATGAGGTTCGCAAGAACC
R delta 19 variant	JB1506	see above
F K43A variant	JB1358	GCGATGCTTCTGTCCGTTTGCCG
R K43A variant	JB1359	CCAGGTATGCTCAGAAAACGCC
F M44A variant	JB1360	GCGCTTCTGTCCGTTTGCCGG
R M44A variant	JB979	TTTCCAGGTATGCTCAGAAAACGCCTG
F K86A variant	JB1367	GCGACTATCCAGCAACATTTGGGC
R K86A variant	JB1368	TACTGCCAGACCGCGC
F Q90A variant	JB1373	GCGCATTTGGGCCAGCTAAACATGC
R Q90A variant	JB1374	CTGGATAGTTTTTACTGCCAGACC
F Q94A variant	JB1686	GCGCTAAACATGCTTCATCGTCGGTC
R Q94A variant	JB1687	GCCCAAATGTTGCTGGATAGTT
F Q90/94A variant	JB1686	see above
F Q90/94A variant	JB1688	GCCCAAATGCGCCTGGATAGTT
F R173A variant	JB1393	GCGATAGCCGAAATTGCCAGGATC
R R173A variant	JB1394	TAACAGGGTGTTATAAGCAATCCC
F E176A variant	JB1397	GCGATTGCCAGGATCAGGGTTAAAG
R E176A variant	JB1398	GGCTATACGTAACAGGGTGTTATAAG
F K244A variant	JB1407	GCGAATGGTGTTGCCGCGC
R K244A variant	JB1408	TCTGACCCGGCAAAACAGG
F R259A variant	JB1411	GCGGCCCTGGAAGGGATTTTTGA
R R259A variant	JB990	AGTTGATAGCTGGCTGGTGGCAGAT
F E262A variant	JB1412	GCGGGGATTTTTGAAGCAACTCATCG
R E262A variant	JB1507	CAGGGCGCGAGTTGATAGCTGGCT
F R282A variant	JB1415	GCGTACCTGGCCTGGTCTGGA
R R282A variant	JB1416	CTGACCAGAGTCATCCTTAGCG
F H289A variant	JB1421	GCGAGTGCCCGTGTCGGA
R H289A variant	JB1422	TCCAGACCAGGCCAGG

Primers used for constructing mammalian expression and reporter vectors LCR assembly, USER cloning, or Golden Gate assembly

Purpose	Name	Sequence
F pCMV sequencing primer	JB697	CGCAAATGGGCGGTAGGCGTG
R pCMV sequencing primer	JB238	TGGTTCTTTCCGCCTCAGAAGC
F pCMV vector	JB247	TAAGCGGCCGCTCGAGCATG
R pCMV vector	JB243	CATGGTGGCTGGATCCGAGCTCGGT
F Tre insert	JB1887	see above
R Tre insert	JB674	GTCGCCATCTTCCAGCAGG
Tre bridging oligo 1	JB1888	ACCGAGCTCGGATCCAGCCACCATGAGCAATTTACTGACCCTGCACCATA
Tre bridging oligo 2	JB1889	CCTGCTGGAAGATGGCGACTAAGCGGCCGCTCGAGCATG
F Brec1 insert	JB1508	see above
R Brec1 insert	JB674	see above
Brec1 bridging oligo 1	JB1910	ACCGAGCTCGGATCCAGCCACCATGAGCATCTTACTGACCCTTCACC
Brec1 bridging oligo 2	JB1889	see above
F pCALNL sequencing primer	JB2267	TCTGCTAACCATGTTCATGCCTTCTTCTTT
R pCALNL sequencing primer	JB2268	CCGTCGACTGCAGAATTCAATTTAAATCGT
F Gibson oligo for BsaI removal	JB2222	GCAATGATACCGCGGGACCCACGCTCACCG
R Gibson oligo for BsaI removal	JB2223	CGGTGAGCGTGGGTCCCGCGGTATCATTGC
F pUC-Kan vector	BT91F	AAGAGCCUGGTTAAAAAATGAGCTGATTTAACAAAAATTTAACGC
R pUC-Kan vector	BT91R	AAGAGCATUCTCGCTCACTGACTCGCTGC
F neo-term insert	JB1945	AATGCTCTUGGTCTCATGCCTGATCAAGAGACAGGATGAGGATCGT
R neo-term insert	JB1946	AGGCTCTUGGTCTCATTGCTGCGTTCGGATTTGATCCAGACA
F Esp3I-BsaI mRFP insert	JB2224	TCGTCTCTGCGTCACTAGGAGACCTAGAAGAGCTATAAACGCAGAAAGGCCCAC
R Esp3I-BsaI mRFP insert	JB2225	ACGTCTCGCGAGTAAGGAGACCAGAAGAGCTCCCTATCAGTGATAGAGATTGAC
F general GG target insert 1	NA	TGGTCTCAACTA[recombinase target]TGCCGGAGACCT
R general GG target insert 1	NA	reverse complement of forward insert 1
F general GG target insert 2	NA	TGGTCTCAGCAA[recombinase target]TTACGGAGACCT
R general GG target insert 2	NA	reverse complement of forward insert 2
F loxP target insert 1	JB1954	TGGTCTCAACTAATAACTTCGTATAGCATACATTATACGAAGTTATTGCCGGAGACCT
R loxP target insert 1	JB1955	AGGTCTCCGGCAATAACTTCGTATAATGTATGCTATACGAAGTTATTAGTTGAGACCA
F general GG target insert 2	JB1956	TGGTCTCAGCAAATAACTTCGTATAGCATACATTATACGAAGTTATTTACGGAGACCT
R general GG target insert 2	JB1957	AGGTCTCCGTAAATAACTTCGTATAATGTATGCTATACGAAGTTATTTGCTGAGACCA

For construction of pCALNL-EGFP *loxP*, *loxLTR*, and *loxBTR* reporter plasmids by Golden Gate, the desired recombinase target was inserted into the general Golden Gate template primer where indicated. Example sequences for inserting the *loxP* target are shown.

Supplementary Methods. Detailed Rec-seq protocol.

An example protocol for an experiment with wild-type Cre and *loxP* oligonucleotides is given. Unless otherwise noted, all enzymes and buffers were purchased from New England Biolabs (NEB), and volumes reflect the standard concentration of the NEB product. All reaction cleanups were performed using the Qiagen Minelute PCR Purification Kit according to manufacturer's instructions.

- *Special care should be taken to avoid nuclease contamination, which will cause anomalous results and ruin the experiment. We suggest using fresh aliquots of commercially-available nuclease-free water and changing aliquots frequently (*e.g.*, weekly, or each time new oligonucleotides are extended).*
- *If recombinase reactions do not proceed to a satisfactory level, the following troubleshooting measures may be attempted:*
    - *Increase protein:DNA molar ratio by half-log increments (*e.g.*, 1:1, 3:1, etc.).*
    - *Increase concentrations of both recombinase and DNA in steps 4-5 by up to 5-fold.*
    - *Supplement reaction buffer with 100 ng BSA and/or 1 μM DTT.*
    - *Undertake more extensive buffer optimization.*

Oligonucleotide extension

1. In a PCR strip, add 15.5 μL nuclease-free water and 2.5 μL NEBuffer 2 to separate wells, one for each oligonucleotide to be extended. For example, to four wells, add 5 μL of a 5 μM stock of library oligonucleotide (*e.g.*, Trans.loxP.1, Trans.loxP.2, Trans.loxPleft.1, and Trans.loxPright.2, one per well).

2. On a thermocycler, run the following program: denature at 95 °C for 3 minutes, then slow cool (ramp at 0.1 °C/s) to 37 °C to anneal the hairpin.

3. Without removing the tubes from the thermocycler, add 1 μL dNTPs and 1 μL Klenow (3'→5' exo-) polymerase to each tube. Let the extension proceed at 37 °C for 1 h. Then, heat-kill the polymerase by incubating at 75 °C for 20 min.

    - *Extended oligonucleotides can be stored at 4 °C for up to one week.*
    - *Extension reactions can be scaled up to double the volume (no need to increase the amount of dNTPs or polymerase, as these are in excess).*

***In vitro* recombination**

4. In a PCR strip, prepare a separate well for each library reaction. In a total reaction volume of 50 μL, add the following: 5 μL NEB Cre recombinase reaction buffer, 1 μL extended left hairpin oligonucleotide, and 1 μL extended right hairpin oligonucleotide.

    - *Each reaction should only contain one randomized library member. For example, combine 1 μL Trans.loxPleft.1 and 1 μL Trans.loxP.2 to investigate mutations in the left half-site of* loxP. *To investigate mutations in the right half-site, combine Trans.loxP.1 and Trans.loxPright.2.*

5. Add recombinase protein to each reaction at the desired ratio. Be sure to include a no-recombinase control reaction. Mix well and incubate at 37 °C for 30 min.

- *For example, our wild-type Cre protein was measured to be 391 µM. For a reaction with a 1:3 protein:DNA ratio, Cre protein was diluted 1184-fold, and 1 µL was added to the reaction.*
- *In general, recombinase protein was diluted to the desired concentration in phosphate-buffered saline (PBS) and then immediately added to the reaction mixture.*

6. Stop each reaction by removing from 37 °C and adding 200 µL Buffer PB. Clean up each reaction using a Qiagen Minelute column, and elute with 55 µL nuclease-free water into a 1.5 mL microcentrifuge tube.

Exonuclease digestion

7. To each tube (including no-recombinase control), add 7 µL NEBuffer 4, 7 µL ATP, and 1 µL each exonuclease I, III, and V (RecBCD). Mix gently and incubate at 37 °C for 45 minutes.

8. Stop each reaction by removing from 37 °C and adding 200 µL Buffer PB. Clean up each reaction using a Qiagen Minelute column, and elute with 20 µL Buffer EB into a 1.5 mL microcentrifuge tube.

Library barcoding and quantification

- *Quantitative PCR (qPCR) is used for several reasons. It confirms that recombinase-treated samples contain more material than no-recombinase controls and no-template controls. It also determines the proper number of cycles to amplify the library DNA during each barcoding step, to avoid biasing the library composition by amplifying past the point of saturation.*

9. Prepare a qPCR master mix for the first barcoding reaction. There will be 2N + 2 reactions of 25 uL each, where N is the number of libraries (for our example, 2 libraries: the left and right half-sites of *loxP*). Each reaction will include the following:
   12.5 µL Universal SYBR Green Supermix (Bio Rad)
   1 µL F.Trans.Miseq primer (10 µM stock)
   1 µL R.Trans.Miseq primer (10 µM stock)
   9.5 µL nuclease-free water
   (leave 1 µL for adding recombinase-treated template)

10. Add the qPCR master mix to wells in a white qPCR strip. For each library reaction, prepare a double-scale well (e.g. 50 µL total, and 2 µL post-recombinase library material as template). Also include 25 µL reactions for no-recombinase control (NRC) and no-template control (NTC).

11. Before running the qPCR reaction, reserve 25 µL from each library reaction in a separate PCR strip.

    - *The 25 µL aliquots will be identical to the sample being run on the qPCR; these reserved reactions will be run on a normal thermocycler once the proper number of amplification cycles has been determined.*

12. Run the qPCR with the following conditions: 98 °C for 30 s, then 34 cycles of (98 °C for 10 s, 57 °C for 5 s, and 72 °C extension for 5 s, followed by a fluorescence reading).

    - *Library samples should reach exponential amplification at least three cycles before NRC and NTC conditions to be considered satisfactorily enriched above background levels.*

13. Based on the qPCR, choose how many cycles to run the reserved primary barcoding samples using the same PCR conditions.

    - *In optimal conditions, i.e. an experiment with wild-type Cre and* loxP *oligonucleotides, Cre-treated samples reach exponential amplification at 22-24 cycles, while NRC and NTC are above 30 cycles. In below-optimal conditions, cycle counts of 28-30 cycles may be observed for recombinase-treated samples.*

14. Clean up each library reaction using Qiagen Minelute columns, and elute in 20 µL Buffer EB.

15. Prepare a qPCR master mix for the secondary barcoding reaction. There will be 2N + 1 reactions of 25 µL each. Each reaction will include the following:
    12.5 µL Universal SYBR Green Supermix
    10 µL nuclease-free water
    (leave 0.5 µL for adding primary barcoded template, 2 µL for primers)

16. Add the qPCR master mix to wells in a white qPCR strip. For each library reaction, prepare a double-scale well (e.g. 47 µL total, and 1 µL post-recombinase library material as template). Each well should also receive 1 µL each of a unique pair of forward and reverse TruSeq Indexing Adapters (10 µM diluted stock, Illumina). Also include a 25 µL NTC reaction.

17. Run the qPCR with the following conditions: 98 °C for 30 s, then 15 cycles of (98 °C for 10 s, 61 °C for 10 s, and 72 °C extension for 5 s, followed by a fluorescence reading).

18. Based on the qPCR, choose how many cycles to run the reserved primary barcoding samples using the same PCR conditions.

    - *Typical secondary-barcoding reactions require less than 10 cycles to reach exponential phase. Reactions should not be run for shorter than 5 cycles, because an excess of un-barcoded material will remain. If the reactions reach exponential phase too quickly, remake the qPCR mix with diluted template.*

19. Clean up each library reaction using Qiagen Minelute columns, and elute in 20 µL Buffer EB.

20. Run an analytical agarose gel with your barcoded library materials and a DNA ladder.

    - *You should observe a single band of approximately 250 bp. If you observe lower MW bands, you will need to gel-extract your sample. Use the Qiagen Gel Extraction Kit according to the manufacturer's instructions.*

21. Combine your library samples and quantify the combined library using a Qubit dsDNA HS Assay Kit (Thermo Fisher Scientific) or KAPA Library Quantification Kit (Roche Sequencing).

22. Run your samples on a MiSeq according to manufacturer's instructions.

Pre-selection library barcoding

- *The first time a synthesized oligonucleotide bearing randomized portions is used, the pre-recombination library must be sequenced in order to calculate enrichment values for post-recombination libraries. The same pre-selection library sequencing data can be used for subsequent experiments using the same oligonucleotide stocks.*

23. Dilute a 100 µM stock of the left-hairpin and right-hairpin synthesized oligonucleotides by 100,000-fold.

24. Prepare a double-scale qPCR reaction for each library oligonucleotide. Each reaction will include the following:
    12.5 µL Universal SYBR Green Supermix
    9.5 µL nuclease-free water
    (leave 1 µL for adding diluted library template, 2 µL for primers)

25. For left-hairpin oligonucleotides (e.g. Trans.loxPleft.1), use primers F.Trans.Miseq and R.presel.Miseq. For right-hairpin oligonucleotides (e.g. Trans.loPright.2), use primers R.Trans.Miseq and F.presel.Miseq. (All primers should first be diluted to 10 µM.)

26. Run the qPCR with the following conditions: 98 °C for 30 s, then 20 cycles of (98 °C for 10 s, 57 °C for 5 s, and 72 °C extension for 5 s, followed by a fluorescence reading).

    - *Typical qPCR reactions require 10-15 cycles to reach exponential phase; redo the qPCR with more or less template if the reactions fall outside this range.*

27. Based on the qPCR, choose how many cycles to run the reserved primary barcoding samples using the same PCR conditions.

28. Cleanup each PCR reaction in 20 µL Buffer EB. Proceed to secondary barcoding, as in step 15 above.

Supplementary References

1. Gibb, B. et al. Requirements for catalysis in the Cre recombinase active site. *Nucleic Acids Res* **38**, 5817-5832 (2010).
2. Karpinski J*, et al.* Directed evolution of a recombinase that excises the provirus of most HIV-1 primary isolates with high specificity. *Nat Biotechnol* **34**, 401-409 (2016).
3. Myung, I.J. Tutorial on maximum likelihood estimation. *J. Math. Psychol.* **47**, 90-100 (2003).
4. de Kok, S. et al. Rapid and reliable DNA assembly via ligase cycling reaction. *ACS Synth Biol* **3**, 97-106 (2014).
