# Supplementary material for: High-resolution specificity profiling and off-target prediction for site-specific DNA recombinases
Source: Nat Commun. 2019 Apr 26;10:1937. doi: 10.1038/s41467-019-09987-0 (PMC6486577; doi:10.1038/s41467-019-09987-0)
Supplement: Supplementary file 2 — Reporting Summary [file 41467_2019_9987_MOESM2_ESM.pdf]

nature research

Corresponding author(s): David R. Liu

Last updated by author(s): April 3, 2019

Reporting Summary

Nature Research wishes to improve the reproducibility of the work that we publish. This form provides structure for consistency and transparency in reporting. For further information on Nature Research policies, see Authors & Referees and the Editorial Policy Checklist.

Statistics

For all statistical analyses, confirm that the following items are present in the figure legend, table legend, main text, or Methods section.

n/a	Confirmed	
☐	☒	The exact sample size (*n*) for each experimental group/condition, given as a discrete number and unit of measurement
☐	☒	A statement on whether measurements were taken from distinct samples or whether the same sample was measured repeatedly
☐	☒	The statistical test(s) used AND whether they are one- or two-sided<br>*Only common tests should be described solely by name; describe more complex techniques in the Methods section.*
☒	☐	A description of all covariates tested
☐	☒	A description of any assumptions or corrections, such as tests of normality and adjustment for multiple comparisons
☐	☒	A full description of the statistical parameters including central tendency (e.g. means) or other basic estimates (e.g. regression coefficient) AND variation (e.g. standard deviation) or associated estimates of uncertainty (e.g. confidence intervals)
☒	☐	For null hypothesis testing, the test statistic (e.g. *F*, *t*, *r*) with confidence intervals, effect sizes, degrees of freedom and *P* value noted<br>*Give P values as exact values whenever suitable.*
☒	☐	For Bayesian analysis, information on the choice of priors and Markov chain Monte Carlo settings
☒	☐	For hierarchical and complex designs, identification of the appropriate level for tests and full reporting of outcomes
☒	☐	Estimates of effect sizes (e.g. Cohen's *d*, Pearson's *r*), indicating how they were calculated

Our web collection on statistics for biologists contains articles on many of the points above.

Software and code

Policy information about availability of computer code

Data collection	No software was used to collect the data.
Data analysis	Custom Python code used for data analysis is available at https://github.com/broadinstitute/rec-seq. BD FACSDiva version 8.0 was used for flow cytometry analysis.

For manuscripts utilizing custom algorithms or software that are central to the research but not yet described in published literature, software must be made available to editors/reviewers. We strongly encourage code deposition in a community repository (e.g. GitHub). See the Nature Research guidelines for submitting code & software for further information.

Data

Policy information about availability of data

All manuscripts must include a data availability statement. This statement should provide the following information, where applicable:

- Accession codes, unique identifiers, or web links for publicly available datasets
- A list of figures that have associated raw data
- A description of any restrictions on data availability

High-throughput sequencing data have been deposited in the NCBI Sequence Read Archive database under accession code PRJNA517947 (SRP182963).

Field-specific reporting

Please select the one below that is the best fit for your research. If you are not sure, read the appropriate sections before making your selection.

☒ Life sciences ☐ Behavioural & social sciences ☐ Ecological, evolutionary & environmental sciences

Life sciences study design

All studies must disclose on these points even when the disclosure is negative.

Sample size	Sequencing depth was chosen to obtain a minimum average of 20,000 reads per Rec-seq experiment based on empirical measurements (using preliminary sequencing) of the library coverage needed for sufficient data quality.
Data exclusions	When calculating enrichment values from sequencing data, recombinase recognition sites with more than 6 mismatches were excluded, as we determined that sequence variants with more than 6 mismatches were typically due to improper alignment caused by sequencing errors (e.g. indels).
Replication	A minimum of 3 independent biological replicates were performed for all experiments.
Randomization	Randomized allocation of cells to experimental groups was not relevant to our study.
Blinding	Blinding was not relevant to our study.

Reporting for specific materials, systems and methods

We require information from authors about some types of materials, experimental systems and methods used in many studies. Here, indicate whether each material, system or method listed is relevant to your study. If you are not sure if a list item applies to your research, read the appropriate section before selecting a response.

Materials & experimental systems

n/a	Involved in the study	
☒	☐	Antibodies
☐	☒	Eukaryotic cell lines
☒	☐	Palaeontology
☒	☐	Animals and other organisms
☒	☐	Human research participants
☒	☐	Clinical data

Methods

n/a	Involved in the study	
☒	☐	ChIP-seq
☐	☒	Flow cytometry
☒	☐	MRI-based neuroimaging

Eukaryotic cell lines

Policy information about cell lines

Cell line source(s)	HEK293T cells were obtained from ATCC (CLR-3216).
Authentication	The cell lines were not independently authenticated because ATCC conducts rigorous authentication.
Mycoplasma contamination	Cell lines were tested every month for mycoplasma contamination and were always negative.
Commonly misidentified lines (See ICLAC register)	No commonly-misidentified cell lines were used in this study.

Flow Cytometry

Plots

Confirm that:

☒ The axis labels state the marker and fluorochrome used (e.g. CD4-FITC).

☒ The axis scales are clearly visible. Include numbers along axes only for bottom left plot of group (a 'group' is an analysis of identical markers).

☒ All plots are contour plots with outliers or pseudocolor plots.

☒ A numerical value for number of cells or percentage (with statistics) is provided.

Methodology

Sample preparation	HEK293T cells were trypsinized, quenched in culture media and run through a flow cytometer.
Instrument	BD Biosciences FACS LSR II HTS

Software	BD Biosciences FACSDiva 8.0
Cell population abundance	No cell sorting was performed.
Gating strategy	Cells were first gated via FSC-A and SSC-A to define the main population. Cells were next gated via FSC-W/FSC-H and SSC-W/SSC-H to define single cells. All cells passing the single-cell gates were included in analysis. Gating for cells positive for both mCherry transfection control (PE Texas Red-A) and EGFP (FITC-A) were determined using negative control populations lack one or both fluorophores.

☒ Tick this box to confirm that a figure exemplifying the gating strategy is provided in the Supplementary Information.
